# Chemotherapeutic induction of cytosolic single-stranded DNA accumulation sensitizes triple-negative breast cancer to immunotherapy

**DOI:** 10.1136/jitc-2025-014722

**Published:** 2026-06-29

**Authors:** Yong Du, Li Yang, Hui Dai, Jianli Zhou, Zhicheng Zhou, Ruoxi Yuan, Rui Ye, Anh Thai Quynh Nguyen, Kishor Bhatia, Shiaw-Yih Lin

**Affiliations:** 1 Systems Biology, The University of Texas MD Anderson Cancer Center, Houston, Texas, USA; 2 Department of Pediatrics, Baylor College of Medicine, Houston, Texas, USA; 3 Lantern Pharma Inc, Dallas, Texas, USA; 4 The University of Texas MD Anderson Cancer Center, Houston, Texas, USA; 5 Hospital for Special Surgery, New York, New York, USA

**Keywords:** Chemotherapy, Immunotherapy, Breast Cancer, Biomarker

## Abstract

**Background:**

Despite the widespread adoption of chemoimmunotherapy in triple-negative breast cancer (TNBC), the mechanisms by which cytotoxic chemotherapy engages antitumor immunity remain poorly defined. Identifying tumor-intrinsic immunogenic programs that predict and enhance responsiveness to immune checkpoint blockade (ICB) is therefore of critical clinical importance.

**Methods:**

Transcriptomic signatures of *TREX1* deficiency were generated from CRISPR-engineered TNBC models and applied to multiple independent TNBC cohorts treated with chemoimmunotherapy. Cytosolic single-stranded DNA (ssDNA) accumulation was quantified using a flow cytometry-based assay to functionally screen chemotherapeutic agents. Immune activation and therapeutic efficacy were evaluated using in vitro assays, syngeneic mouse tumor models, flow cytometry, and single-cell RNA sequencing. Statistical analyses included two-sided t tests or Wilcoxon tests, analysis of variance where appropriate, and receiver operating characteristic analyses.

**Results:**

An in vivo TREX1-deficiency transcriptional signature, reflecting ssDNA-driven immune programs rather than *TREX1* expression alone, robustly predicted clinical response to chemoimmunotherapy across independent TNBC cohorts. Functional screening identified LP-184, an acylfulvene-derived alkylating agent in clinical development, as a potent inducer of cytosolic ssDNA and type I interferon signaling. LP-184 enhanced antigen presentation, reduced immunosuppressive M2-like macrophages, promoted CD8^+^ T-cell priming, and synergized with anti-programmed cell death protein 1 therapy in vivo. Exogenous ssDNA recapitulated key immunostimulatory effects of LP-184, supporting ssDNA accumulation as a central mechanistic mediator.

**Conclusions:**

These findings establish cytosolic ssDNA-driven immune programs as a mechanistic link between DNA damage and antitumor immunity, providing both a predictive biomarker and a therapeutic axis for chemoimmunotherapy. Pharmacologic induction of ssDNA represents a rational strategy to enhance ICB efficacy and expand immunotherapy responsiveness in TNBC.

**WHAT IS ALREADY KNOWN ON THIS TOPIC**

- Cytotoxic chemotherapy can enhance antitumor immunity and improve responses to immune checkpoint blockade, but the tumor-intrinsic mechanisms linking DNA damage to immune activation remain poorly defined. In particular, reliable biomarkers and actionable pathways that explain why certain chemotherapies synergize with immunotherapy are lacking.

**WHAT THIS STUDY ADDS**

- This study identifies cytosolic single-stranded DNA (ssDNA) accumulation as a mechanistic driver of chemotherapy-induced antitumor immunity. Using genetic and pharmacologic approaches, we show that the acylfulvene-derived agent LP-184 potently induces cytosolic ssDNA, activates type I interferon signaling, remodels the myeloid tumor microenvironment, and sensitizes triple-negative breast cancer to immune checkpoint blockade.

**HOW THIS STUDY MIGHT AFFECT RESEARCH, PRACTICE OR POLICY**

- These findings establish cytosolic ssDNA as both a functional biomarker and a therapeutic axis linking chemotherapy to immunotherapy efficacy. Pharmacologic induction of ssDNA may inform rational chemoimmunotherapy combinations and guide the development of next-generation immunogenic chemotherapeutic strategies.

## Introduction

 Triple-negative breast cancer (TNBC) remains a major clinical challenge due to the lack of actionable molecular targets and poor prognosis.^1^ Chemotherapy remains the mainstay of treatment, yet responses are limited and relapse is common.^1^ Immune checkpoint blockade (ICB) has shown encouraging activity in a subset of TNBCs—particularly those with PD-L1 expression or high tumor-infiltrating lymphocytes—but most patients fail to respond.^1^ This underscores a growing interest in developing new chemotherapeutic agents that retain the cytotoxic efficacy of conventional chemotherapy while also enhancing tumor immunogenicity to potentiate ICB response.^2 3^ Biomarkers that can reliably guide the development of such agents as well as predict the efficacy of chemotherapy and immunotherapy combinations remain critically needed.

DNA damage can generate cytosolic nucleic acids through multiple mechanisms, including replication stress, micronuclei formation and rupture, and defective DNA repair, leading to activation of innate immune sensing pathways. These nucleic acids are primarily detected by the cyclic GMP-AMP synthase (cGAS)–stimulator of interferon genes (STING) pathway.^4^,^7^ Within this framework, nucleases such as TREX1 act as critical regulators by degrading cytosolic DNA and limiting aberrant immune activation, as highlighted by its role in autoimmune diseases such as Aicardi-Goutières syndrome and systemic lupus erythematosus.^4 8 9^ TREX1 deficiency has been strongly linked to autoimmune and inflammatory disorders, where accumulation of self-DNA drives chronic type I interferon (IFN-I) responses.^8^ These findings highlight a conserved mechanism by which dysregulated nucleic acid metabolism can bridge DNA damage and immune activation across diverse disease contexts.^4^

Recent studies, including ours, have revealed that cytosolic single-stranded DNA (ssDNA) accumulation acts as a potent immunostimulatory signal that activates IFN-I pathways and contributes to ICB responsiveness.^10^,^15^ The exonuclease TREX1 is a key negative regulator of this process, degrading aberrant cytosolic ssDNA to prevent chronic immune activation.^9 16 17^ By integrating transcriptomic profiles from *TREX1*-deficient tumors and experimental models, we established an in vivo TREX1-deficiency signature that strongly predicts chemoimmunotherapy responsiveness across independent TNBC cohorts. This finding supports the concept that pharmacologic ssDNA accumulation—mimicking *TREX1* loss—could serve as a mechanistically grounded approach to enhance immunotherapy efficacy. In contrast, *TREX1* expression alone does not distinguish responders from non-responders.

Mechanistically, cytosolic ssDNA levels reflect a balance between its generation (eg, replication stress and DNA damage) and its degradation by nucleases such as TREX1. Inhibiting TREX1 blocks ssDNA degradation but does not generate new ssDNA; thus, the immunogenic effect of TREX1 inhibition likely depends on the tumor’s basal ssDNA level. In contrast, DNA-damaging chemotherapeutics can directly induce ssDNA formation, providing an alternative or complementary route to promote cytosolic ssDNA accumulation—especially in ssDNA-low tumors. This rationale suggests that identifying ssDNA-inducing chemotherapies may represent a more practical and druggable strategy than enzymatic inhibition of TREX1 alone, offering the dual advantage of preserving the antitumor potency of conventional chemotherapy and enhancing responsiveness to immunotherapy.

Guided by this concept, we developed a flow cytometry-based quantitative platform to assess cytosolic ssDNA levels in drug-treated TNBC cells, enabling functional screening for compounds that promote ssDNA accumulation. Using this approach, we evaluated a panel of chemotherapeutic agents (National Comprehensive Cancer Network (NCCN)-recommended and agents in development) and identified paclitaxel, doxorubicin, and LP-184, a novel alkylating agent in early clinical development, as potent pharmacologic inducers of ssDNA accumulation. LP-184 treatment activated IFN-I signaling in a cGAS-dependent manner, enhanced antigen presentation, and reprogrammed the tumor microenvironment (TME) toward an immunostimulatory state.

In summary, our study introduces an ssDNA-centered framework that unifies genetic (*TREX1* deficiency) and pharmacologic (ssDNA-inducing drugs) mechanisms of immune activation. It establishes a *TREX1-*deficiency signature as a predictive biomarker for chemoimmunotherapy response and identifies LP-184 as a pharmacologic mimic of *TREX1* loss that bridges DNA damage and immune activation, offering a promising strategy for chemotherapeutic drug development to sensitize TNBC to immunotherapy.

## Results

### An in vivo TREX1-deficiency signature, rather than *TREX1* expression, predicts chemoimmunotherapy response in TNBC

TNBC remains largely dependent on chemotherapy, and the clinical benefit of ICB is limited to a subset of patients.^1^ Combination therapy with chemotherapy and programmed cell death protein (PD-1) blockade has now become a promising regimen for selected TNBC populations,^2 3^ underscoring the importance of identifying tumor-intrinsic determinants that modulate chemo-ICB responsiveness. To this end, we focused on the DNA exonuclease *TREX1*, an endoplasmic reticulum-anchored 3′→5′ exonuclease that clears cytosolic ssDNA and restrains DNA sensing and IFN-I signaling. While we and others have reported that inhibition or loss of *TREX1* enhances IFN-I responses and sensitizes tumors to anti-PD-1 therapy,^10^,^15^ it remains unclear how to capture TREX1-related immunostimulatory programs in a way that predicts clinical outcomes.

**Figure 1:**
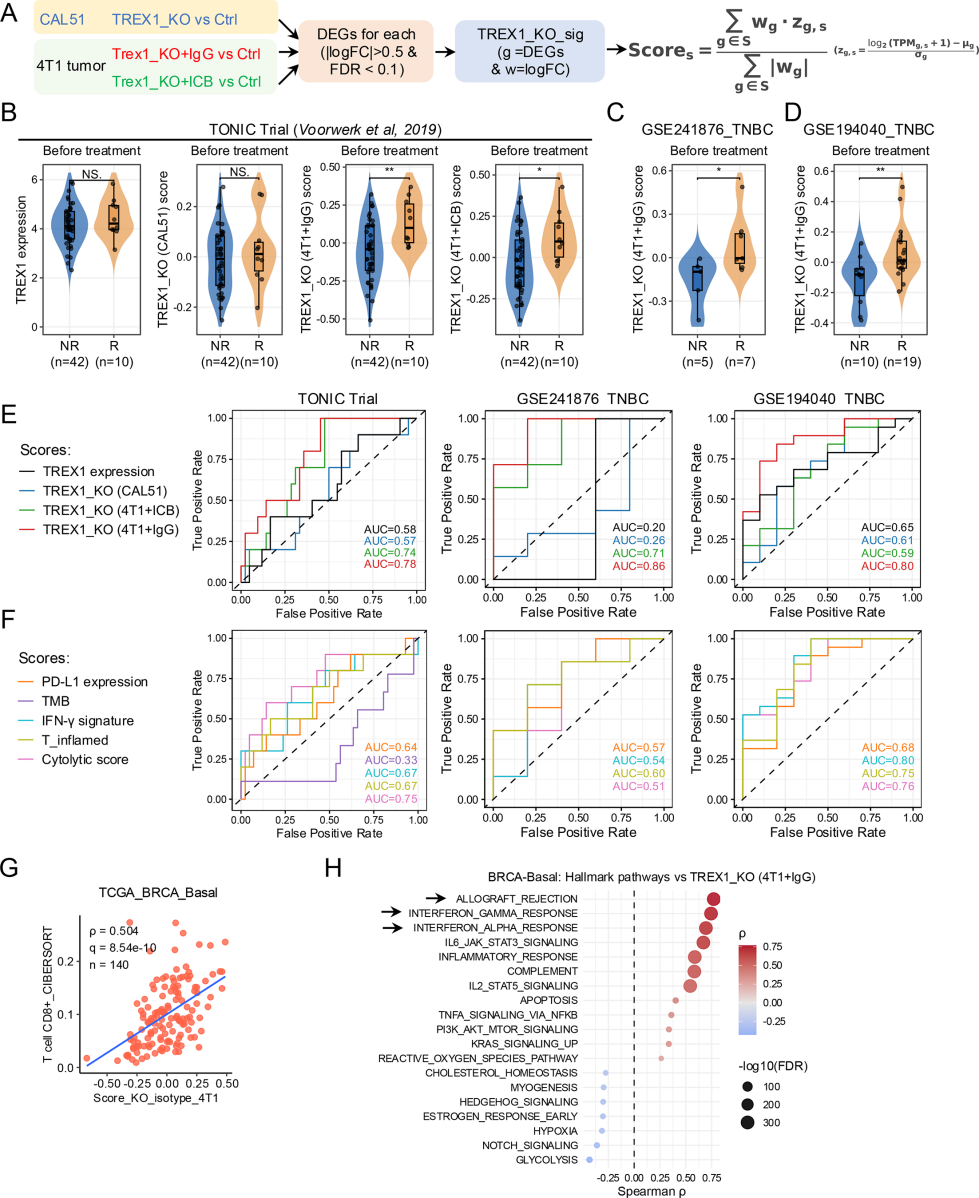
An in vivo TREX1-deficiency signature, rather than *TREX1* expression, predicts chemoimmunotherapy response in TNBC. (**A**) Design and computation of the in vitro and in vivo TREX1 knockout (TREX1_KO) signatures. Differential expression analysis was performed on RNA-seq datasets from CAL51 (*TREX1*-KO vs Ctrl) and 4T1 tumors (*Trex1*-KO+IgG vs WT+IgG; *Trex1*-KO+ICB vs WT+IgG), using thresholds of |log₂FC|>0.5 and FDR<0.1. Mouse genes in the 4T1 models were converted to their human orthologs. Each analysis yielded a set of differentially expressed genes (DEGs) with associated log₂FC values. Each DEG set was used to define a context-specific *TREX1_KO* signature, with gene-specific weights corresponding to their log₂FC values. For each sample, the signature score was calculated as Score = Σ(w₍g₎ × z₍g₎) / Σ|w₍g₎|, where w₍g₎ is the gene weight (log₂FC) and z₍g₎ is the per-gene z-score of log₂(TPM+1). (B–D) Violin/box overlays showing the indicated signature scores in non-responders (NR) versus responders (R) at pre-treatment in TONIC (**B**) multiple chemotherapy+anti-programmed cell death protein (PD-1), GSE241876 (**C**) Carboplatin+Nab-Paclitaxel + anti-PD1 and GSE194040 (**D**) Paclitaxel+anti-PD1 TNBC datasets. Central dot indicates the median. P values were determined by two-sided Wilcoxon rank-sum test. Group sizes are shown below each panel. (E–F) ROC analyses of *TREX1*-related readouts (**E**) and benchmark predictors (**F**) at baseline across cohorts. Curves and corresponding AUCs are shown for TREX1 expression, *TREX1*_KO (CAL51), *TREX1*_KO (4T1+ICB), *TREX1*_KO (4T1+IgG), PD-L1 expression, TMB, IFN-γ signature, and T cell–inflamed and cytolytic scores, colored as indicated. The dashed line denotes random performance. (G–H) Correlative analyses in TCGA BRCA basal-like tumors. (**G**) Correlation between the *TREX1*_KO (4T1+IgG) score and CD8^+^ T-cell abundance estimated by CIBERSORT. Spearman ρ and adjusted p values are shown. (**H**) Dot plot showing Spearman correlations between Hallmark ssGSEA pathway scores and the *TREX1*_KO (4T1+IgG) score; color encodes ρ and point size encodes −log₁₀(FDR). Only pathways meeting display thresholds (|ρ| > 0.2, FDR<0.05) are shown. AUC, area under the curve; IFN, interferon; IgG, immunoglobulin G; ROC, receiver-operating characteristic; TNBC, triple-negative breast cancer.

To address this, we generated CAL51 *TREX1*-knockout (KO) and 4T1 *Trex1*-KO cell lines using CRISPR/Cas9 editing. The 4T1 *Trex1*-KO line was subsequently implanted into syngeneic BALB/c mice and treated with either control immunoglobulin G (IgG) or anti-PD-1 antibody for 1 week.^10^ These experimental settings enabled us to derive three context-specific *TREX1*-deficiency transcriptional signatures, representing tumor-intrinsic effects in vitro as well as tumor-context responses in vivo with or without ICB treatment. RNA sequencing was performed on both the in vitro KO models and the in vivo tumor samples. Differential expression analysis identified gene sets consistently induced by *TREX1/Trex1* loss (online supplemental figure S1A–C). These differentially regulated genes were integrated to construct in vitro and in vivo TREX1-deficiency signatures (figure 1A), in which genes were weighted by their log₂ fold-change (online supplemental figure S1A–C), representing the transcriptional footprint of ssDNA-driven immune activation. Samples were scored as the weighted sum of row-wise z-scored log₂-transformed expression (figure 1A).

Across TNBC biopsies, the *TREX1*_KO (4T1+IgG) score was significantly higher in chemoimmunotherapy responders than in non-responders across three independent TNBC cohorts (TONIC trial,^18^ GSE241876,^19^ GSE194040^20^), whereas signatures derived from in vitro CAL51 cells or *TREX1*_KO (4T1+ICB) tumors showed minimal separation between outcome groups (figure 1B–D; online supplemental figure S1D and E). Notably, the TONIC study uses an induction design prior to ICB, whereas the other cohorts represent chemoimmunotherapy combination settings, indicating that the predictive performance of the TREX1_KO signature is robust across different treatment schedules. In receiver operating characteristic analyses, the *TREX1*_KO (4T1+IgG) signature consistently outperformed *TREX1* expression and other KO-derived signatures across all cohorts (figure 1E). In contrast, TREX1 mRNA expression alone failed to predict clinical outcomes across these datasets (figure 1A,E; online supplemental figure S1D and E). Neither *TREX1* expression (AUC=0.20–0.65) nor the *TREX1*_KO (CAL51) signature (0.26–0.61) showed meaningful predictive capacity. The *TREX1*_KO (4T1+ICB) signature also demonstrated moderate predictive ability (AUC=0.59–0.74), but its overall performance was lower than that of the in vivo *TREX1*_KO (4T1+IgG) signature, underscoring the importance of in vivo context in capturing the immunostimulatory programs linked to chemoimmunotherapy responsiveness. When benchmarked against established biomarkers—including PD-L1 expression (AUC=0.57–0.68), tumor mutational burden (TMB, 0.33), IFN-γ signatures (0.54–0.80), T cell–inflamed signatures (0.60–0.75), and the cytolytic score (0.51–0.76)—the *TREX1*_KO (4T1+IgG) signature demonstrated comparable or superior discrimination of responders, achieving AUCs of 0.78–0.86 across independent datasets (figure 1E,F).

Mechanistically, higher TREX1_KO_ (4T1+IgG) scores in TNBC tumors correlated positively with CD8^+^ T-cell infiltration (figure 1G; online supplemental figure S1F and H) and with Hallmark pathways related to allograft rejection and type I/II IFN responses (figure 1H; online supplemental figure S1G and I). Together, these data indicate that a transcriptional footprint of *TREX1* deficiency—rather than *TREX1* expression per se—captures an IFN-high, inflamed tumor context associated with enhanced chemo-immunotherapy responsiveness in TNBC.

### Chemotherapeutic screening identifies LP-184 as a potent inducer of cytosolic ssDNA

The above findings establish that the immunostimulatory transcriptional program elicited by *TREX1* loss robustly predicts response to chemoimmunotherapy in TNBC. Rather than reflecting *TREX1* expression itself, this signature captures a downstream ssDNA-induced immune program, indicating that cytosolic ssDNA accumulation serves as a functional determinant of tumor immunogenicity. Pre-treatment tumors with higher *TREX1*-KO signature scores likely possess greater intrinsic ssDNA-generating capacity, enabling stronger innate immune activation when genotoxic chemotherapy further elevates ssDNA levels, whereas ssDNA-low tumors may remain immunologically inert until sufficient ssDNA is induced. We therefore hypothesized that pharmacologic induction of cytosolic ssDNA could recapitulate the immune-activating effects of *TREX1* deficiency, thereby linking DNA damage to immune activation.

**Figure 2:**
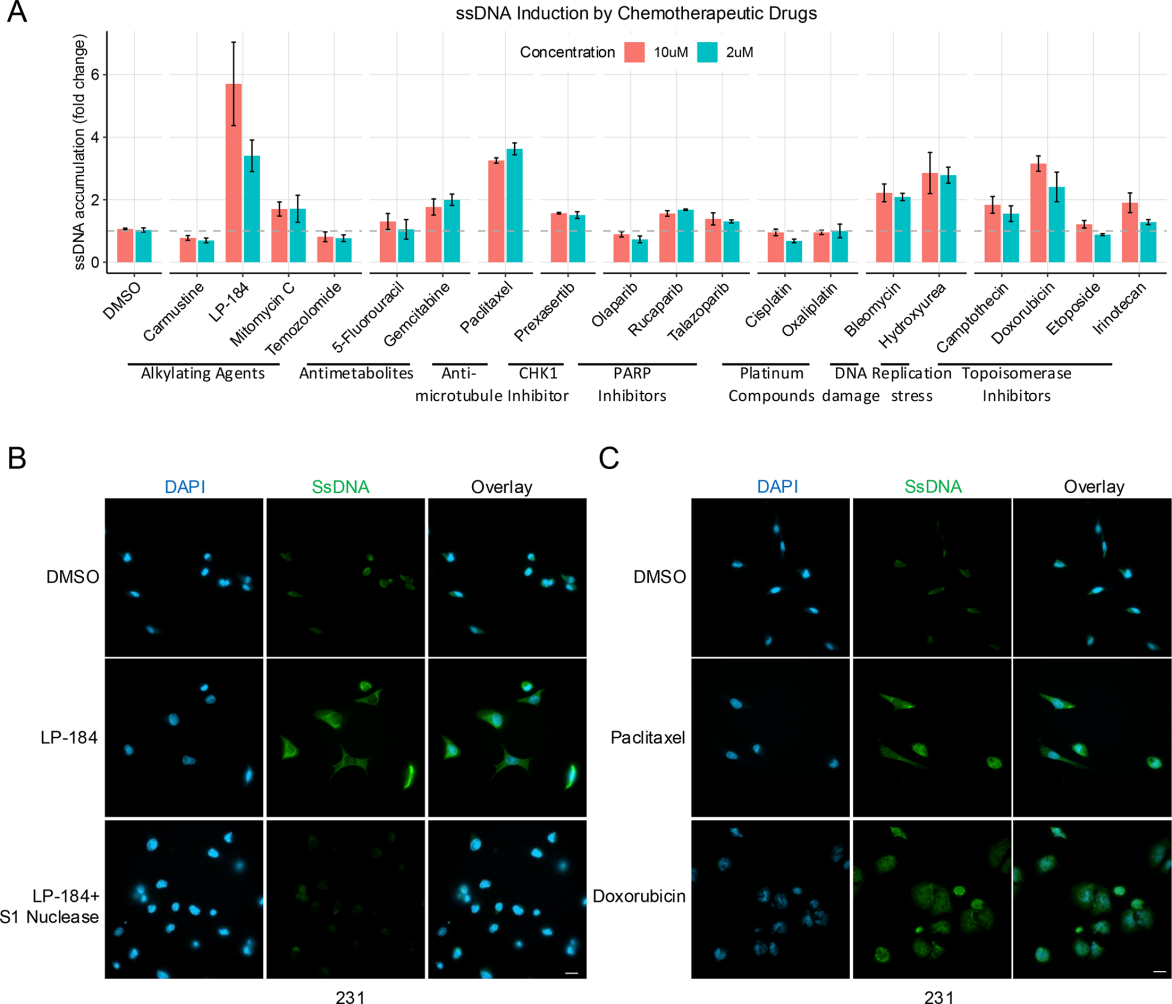
Chemotherapeutic drug screening identifies LP-184 as a potent inducer of single-stranded DNA (ssDNA). (**A**) The histogram plot of ssDNA induction (fold change normalized to DMSO) by chemotherapeutic agents tested at 10 µM and 2 µM for 48 hours in 231 cells. Hydroxyurea was run at 10 mM and 2 mM as a positive control. Bars show mean±SEM, n=3 independent experiments. (B–C) Immunofluorescence staining of 231 cells showing cytosolic ssDNA (green) and nuclei (blue). (**B**) Representative images of cells treated with DMSO, LP-184 (0.4 µM), or LP-184 plus S1 nuclease. (**C**) Representative images of cells treated with paclitaxel (0.4 µM) or doxorubicin (0.4 µM). Scale bar, 20 µm.

This concept may open new therapeutic avenues for TNBC, in which chemotherapy not only remains the backbone of treatment but can also be leveraged to enhance immune responsiveness. To test this hypothesis, we established a quantitative flow-cytometric assay for cytosolic ssDNA using a well-validated ssDNA-specific antibody^21^,^24^ and performed a focused screen of chemotherapeutic agents to identify compounds capable of inducing ssDNA accumulation and activating type I IFN signaling. We screened 19 chemotherapeutic agents spanning the major NCCN classes—including alkylating agents, platinum compounds, antimetabolites, topoisomerase inhibitors, PARP and CHK1 inhibitors, antimicrotubule agents, and ROS inducers—at 10 µM and 2 µM for 48 hours in MDA-MB-231 (hereafter, 231) cells. Cytosolic ssDNA was quantified using the anti-ssDNA flow assay (figure 2A). Among all tested drugs, LP-184, an acylfulvene-derived alkylating agent that has demonstrated safety in phase I/II clinical development (*NCT05933265*), ranked among the strongest ssDNA inducers, increasing cytosolic ssDNA levels by approximately sixfold at 10 µM and 3.5-fold at 2 µM relative to vehicle. In contrast, most other agents produced minimal changes (figure 2A). Notably, paclitaxel and doxorubicin—two standard TNBC chemotherapies—also elicited marked ssDNA accumulation (figure 2A) and are both known to synergize with ICB assay.^2 25 26^ We next used immunofluorescence to further validate cytosolic ssDNA accumulation following LP-184 treatment (figure 2B) as well as paclitaxel and doxorubicin treatment (figure 2C).

### LP-184 induces greater ssDNA accumulation than genetic ablation of *TREX1*

**Figure 3:**
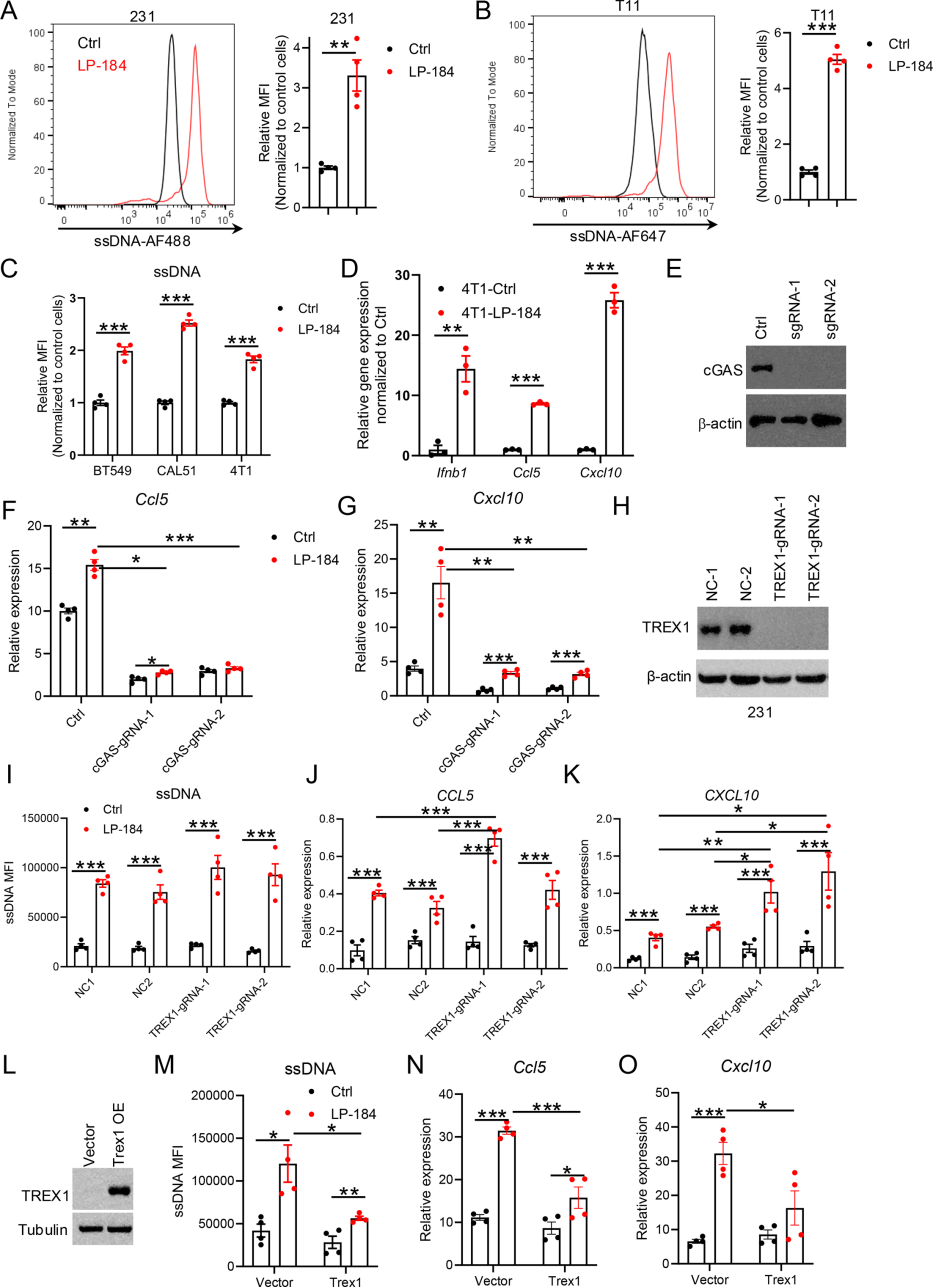
LP-184 induces greater single-stranded DNA (ssDNA) accumulation and cytokine expression than genetic ablation of *TREX1*. (A–B) Flow-cytometric analysis of cytosolic ssDNA in 231 (A, 2 µM) and T11 (B, 10 µM) cells. Left, representative histograms (red, LP-184; black, control). Right, mean fluorescence intensity (MFI) normalized to each experiment’s respective control; mean±SEM, n=4; two-sided t-test,**p<0.01, ***p<0.001. (**C**) Quantification of ssDNA by flow cytometry in BT-549 (2 µM), CAL-51 (2 µM), and 4T1 (10 µM) cells treated with LP-184 or DMSO. MFI values were normalized to each cell line’s respective control; mean±SEM, n=4; two-sided t-test, ***p<0.001. (**D**) qRT–PCR analysis of *Ifnb1*, *Ccl5*, and *Cxcl10* expression in 4T1 cells treated with LP-184. Data represent mean±SEM, n=3; two-sided t-test; *p<0.05, **p<0.01, ***p<0.001. (**E**) Immunoblot analysis confirming efficient knockout of cGAS in 4T1 cells using two independent sgRNAs. (F–G) Relative expression of *Ccl5* (**F**) and *Cxcl10* (**G**) in cGAS-deficient 4T1 cells following LP-184 treatment, measured by qRT-PCR. (**H**) Immunoblot analysis confirming TREX1 knockout in 231 cells using two independent sgRNAs. (**I**) Flow-cytometric quantification of cytosolic ssDNA levels in control (NC) and TREX1 knockout 231 cells, with or without LP-184 treatment. (J–K) Relative expression of *CCL5* (**J**) and *CXCL10* (**K**) in control and TREX1 knockout 231 cells following LP-184 treatment. (**L**) Immunoblot analysis confirming mouse TREX1 overexpression in 4T1 cells. (**M**) Flow-cytometric quantification of cytosolic ssDNA levels in vector control and Trex1-overexpressing cells with or without LP-184 treatment. (N–O) Relative expression of *Ccl5* (**N**) and *Cxcl10* (**O**) in vector control and Trex1-overexpressing cells following LP-184 treatment. Data are presented as mean±SEM. Statistical significance was determined by two-sided unpaired Student’s *t*-test. *p<0.05, **p<0.01, ***p<0.001.

To confirm that ssDNA induction by LP-184 is broadly applicable, we examined additional TNBC models and observed robust ssDNA induction across multiple cell lines, including human (231, BT-549, CAL-51) and murine (T11, 4T1) cells (figure 3A–C). Next, we confirmed by qPCR that LP-184 treatment strongly induces an IFN-I–associated transcriptional program, including *Ifnb1*, *Ccl5*, and *Cxcl10* (figure 3D). Genetic ablation of cGAS significantly attenuated the induction of *Ccl5* and *Cxcl10*, indicating that LP-184–induced inflammatory signaling is largely dependent on the cGAS pathway (figure 3E–G).

To directly compare pharmacologic induction of ssDNA with impaired ssDNA degradation, we generated *TREX1*-deficient 231 cells by co-electroporating Cas9 protein with *TREX1* gRNAs. Efficient depletion of TREX1 protein was confirmed by Western blot (figure 3H). *TREX1* loss resulted in a modest increase in cytosolic ssDNA levels that was most apparent at early time points following genome editing but progressively diminished on passaging (figure 3I), likely reflecting cellular adaptation or selection against sustained nucleic acid accumulation. Importantly, even at its peak, the magnitude of ssDNA accumulation on *TREX1* loss remained substantially lower than that induced by LP-184. Moreover, *TREX1* loss mildly elevated *CXCL10* expression, whereas LP-184 treatment further amplified this response, with a trend toward enhanced induction in *TREX1*-deficient cells. These findings suggest that ssDNA accumulation induced by LP-184 exceeds the threshold required for maximal activation of downstream innate immune signaling (figure 3J,K). Finally, overexpression of Trex1 partially reduced LP-184–induced ssDNA accumulation and attenuated downstream inflammatory gene expression, further supporting a central role for cytosolic ssDNA in mediating LP-184–driven immune activation (figure 3L–O).

Together, these findings demonstrate that LP-184 induces cytosolic ssDNA accumulation to a level that surpasses that achieved by genetic loss of TREX1, thereby robustly activating cGAS-dependent innate immune signaling in TNBC cells.

### LP-184 and ssDNA enhance anti-PD-1 efficacy by increasing effector T cell responses and reducing M2 macrophages *in vivo*

Given that LP-184 robustly induced cytosolic ssDNA and activated type I IFN signaling in vitro, we next evaluated whether these effects could translate into improved antitumor immunity in vivo. To test this, we examined the impact of LP-184 or exogenous ssDNA delivery on tumor growth, immune composition, and effector T-cell activation in syngeneic TNBC models.

**Figure 4:**
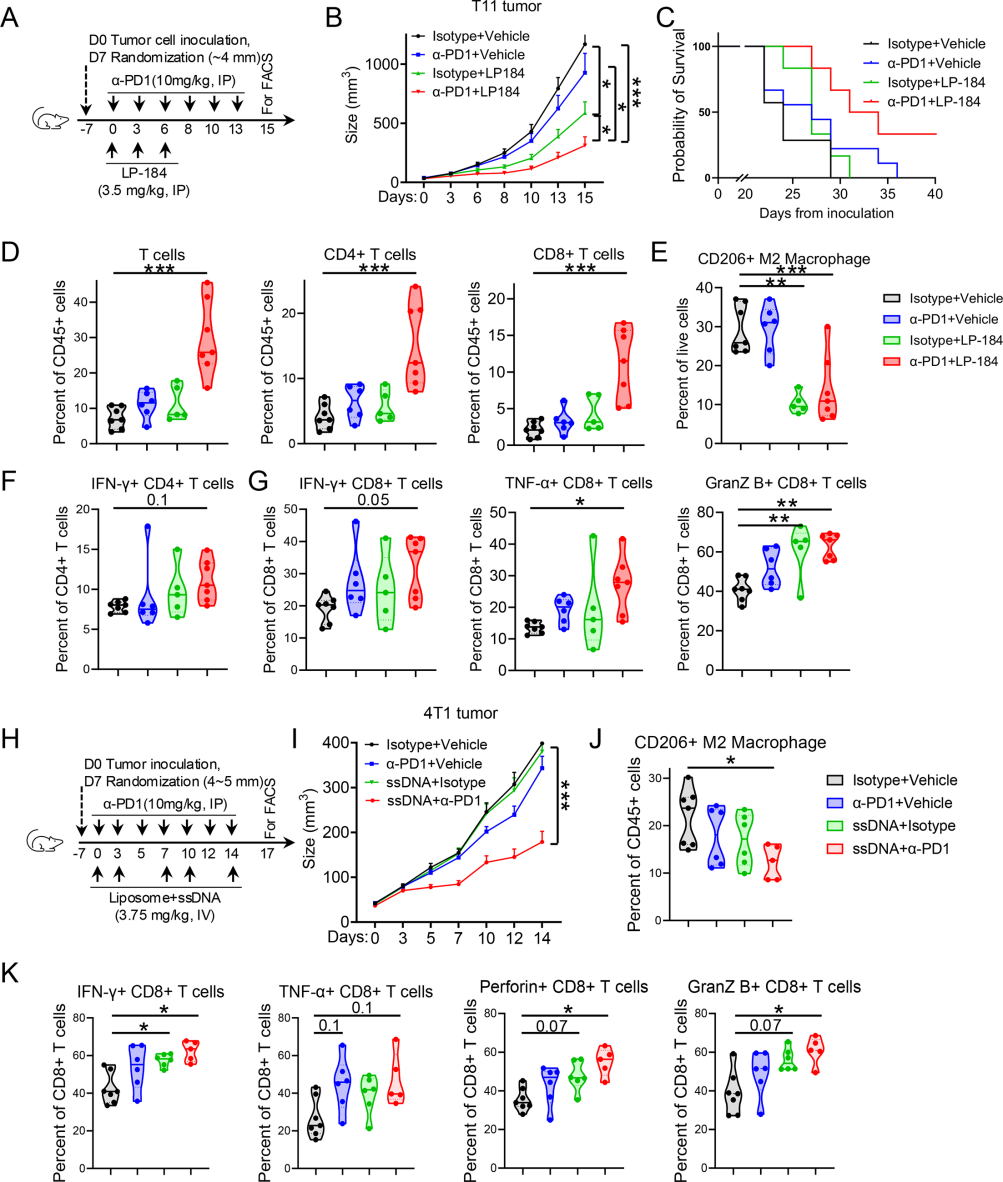
LP-184 and single-stranded DNA (ssDNA) enhance anti–PD-1 efficacy by increasing CD8 T-cell cytotoxic function and reducing M2 macrophages in vivo. (**A**) Experimental design for LP-184±anti–PD-1 therapy (**T11**). **(**B****), T11 tumor growth curves. Groups: isotype + vehicle (n=12), α-PD-1 + vehicle (n=13), isotype + LP-184 (n=12), α-PD-1 + LP-184 (n=13). (**C**), Kaplan-Meier survival analysis for the same treatment groups as in B. (D–G) Flow-cytometric analysis of immune composition and T-cell cytotoxic function in T11 tumors: immune-cell composition of T cells (**D**, left), CD4^+^ T cells (**D**, middle), CD8^+^ T cells (**D**, right), and CD206^+^ (M2-like) macrophages (**E**) among CD45^+^ cells; IFN-γ^+^ CD4^+^ T cells among CD4^+^ T cells (**F**), IFN-γ^+^ CD8^+^ T cells, TNF-α^+^ CD8^+^ T cells, and Granzyme B^+^ CD8^+^ T cells among CD8^+^ T cells (**G**). **(**H****) Experimental design for ssDNA delivery combined with anti–PD-1 therapy (4T1). (**I**) 4T1 tumor growth curves. (J–K) Flow-cytometric analysis of immune composition and T-cell cytotoxic function in 4T1 tumors: CD206^+^ (M2-like) macrophages among CD45^+^ cells (**J**); IFN-γ^+^ CD8^+^ T cells, TNF-α^+^ CD8^+^ T cells, Perforin^+^ CD8^+^ T cells, and Granzyme B^+^ CD8^+^ T cells among CD8^+^ T cells (**K**). Data are mean±SEM; significance symbols: * p<0.05; ** p<0.01; *** p<0.001. Statistical tests: two-way analysis of variance (ANOVA) with Dunnett’s multiple comparisons at endpoint (**B, I**) and (**D–F, J and K**). IFN, interferon.

To determine whether LP-184 could potentiate ICB in vivo, we evaluated its combination with anti-PD-1 in syngeneic T11 and 4T1 tumor models. In both models, combination therapy significantly suppressed tumor growth compared with either monotherapy (figure 4A–C, online supplemental figure S2A-C) while having only a minor impact on body weight (online supplemental figure S2D). We next examined how the TME was altered. In T11 tumors, flow cytometry revealed that combination treatment markedly increased intratumoral T-cell infiltration, including both CD4^+^ and CD8^+^ T cells, while reducing immunosuppressive CD206^+^ M2-like macrophages (figure 4D–E, online supplemental figure S3A). Functionally, combination therapy significantly enhanced T-cell cytotoxicity activity, with increased frequencies of IFN-γ^+^ CD4^+^ (figure 4F), IFN-γ^+^, TNF-α,^+^ and granzyme B^+^ CD8^+^ T cells (figure 4G, online supplemental figure S3B).

Because LP-184 treatment induces tumor-derived ssDNA, we asked whether exogenous ssDNA could reproduce its immunostimulatory activity. In the 4T1 model, combining ssDNA with anti-PD-1 significantly inhibited tumor growth compared with either treatment alone (figure 4H,I, online supplemental figure S2E and F). This effect was accompanied by a reduction of CD206^+^ M2-like macrophages (figure 4J) and increased frequencies of cytotoxic T-cell populations, including IFN-γ^+^, TNF^+^, granzyme B,^+^ and perforin^+^ CD8^+^ T cells (figure 4K). These data indicate that ssDNA can recapitulate the key immune features observed with LP-184, supporting ssDNA as a mechanistic mediator of LP-184–driven potentiation of anti–PD-1 therapy.

### LP-184–treated cancer cells induce macrophage anti-tumor function

**Figure 5:**
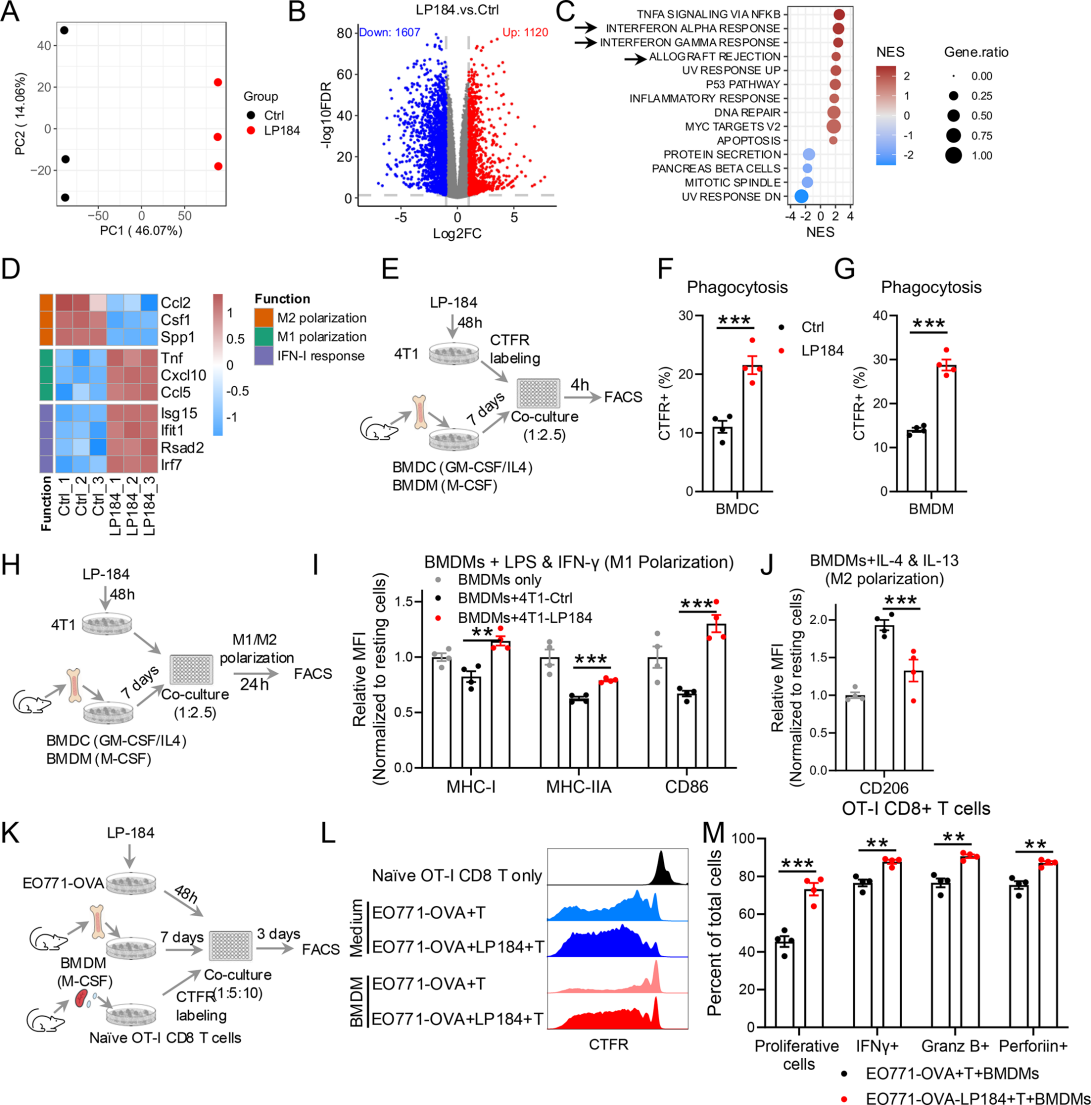
LP-184-treated cancer cells induce macrophage anti-tumor function. (**A**) Principal component analysis (PCA) of RNA-seq profiles from 4T1 cells treated with LP-184 versus control. (**B**) Volcano plot of differentially expressed genes in LP-184-treated versus control 4T1 cells (|log₂FC|>1, FDR<0.05). (**C**) Gene set enrichment analysis of upregulated genes in LP-184-treated cells showing enrichment in type I/II interferon response and allograft rejection signaling pathways. (**D**) Heatmap of representative upregulated genes grouped by immune function (M1/M2 polarization and IFN-I response). (**E**) Experimental design for co-culture of LP-184-treated 4T1 cells with GM-CSF/IL-4-differentiated BMDCs or M-CSF-differentiated BMDMs. 4T1 cells were pre-treated with LP-184 for 48 hours, labeled with CTFR dye, and co-cultured with BMDCs or BMDMs for 4 hours before flow cytometry. (F–G) Flow-cytometric quantification of phagocytosis by BMDCs (**F**) and BMDMs (**G**); mean±SEM, n=4. (**H**) Experimental design for macrophage polarization assays: 4T1 cells were pre-treated with LP-184 for 48 hours and co-cultured with BMDMs or BMDCs under M1 (LPS+IFN-γ) or M2 (IL-4+IL-13) conditions. (I–J) Flow-cytometric quantification of macrophage polarization markers after co-culture: MHC-I, MHC-IIA, and CD86 (M1 markers); **(I**) CD206 (M2 marker); **(J**) mean fluorescence intensity (MFI) normalized to control; mean±SEM, n=4. (**K**) Experimental design for OT-I T-cell priming assay. LP-184–treated EO771-OVA tumor cells were co-cultured with BMDMs and CTFR-labeled naïve OT-I CD8^+^ T cells for 3 days. (**L**) Representative flow-cytometric histograms showing proliferation of OT-I CD8^+^ T cells under indicated co-culture conditions. (**M**) Flow-cytometric quantification of proliferative (CTFR low) and activated OT-I CD8^+^ T cells expressing IFN-γ, granzyme B, and perforin; mean±SEM, n=4. Statistical analysis: two-sided t-test; significance symbols: p<0.05, *p<0.01, **p<0.001. BMDC, bone-marrow-derived dendritic cells; BMDM, bone-marrow-derived macrophage; CTFR, CellTrace Far Red; IFN, interferon.

To investigate how LP-184-induced tumor stress enhances immune activation, we performed transcriptomic profiling of LP-184-treated 4T1 cells (figure 5A–D). Principal component analysis revealed a marked transcriptomic shift following LP-184 treatment (figure 5A). Differential gene-expression analysis identified broad upregulation and downregulation of immune-related genes (figure 5B). Pathway enrichment analysis of Hallmark gene sets showed strong activation of anti-tumor programs, including type I/II IFN responses, allograft rejection, and immunostimulatory chemokine signaling (figure 5C), consistent with the *TREX1*-KO-associated IFN-I signature described earlier. Moreover, LP-184 treatment upregulated genes associated with M1 macrophage polarization while reducing those linked to M2 polarization (figure 5D), prompting us to examine how LP-184-treated tumor cells influence DC, macrophage, and T-cell functions.

We next assessed whether these transcriptional changes could reprogram innate immune activity. LP-184-treated tumor cells were co-cultured with bone-marrow-derived dendritic cells (BMDCs) or macrophages (BMDMs) labeled with CellTrace Far Red dye to assess phagocytic uptake (figure 5E). Flow-cytometric analysis showed significantly increased engulfment of LP-184–treated tumor cells by both BMDCs and BMDMs (figure 5F,G, (online supplemental figure S3C and D), indicating enhanced immunogenic recognition.

To determine whether LP-184–treated tumor cells modulate macrophage polarization, we performed co-culture assays under M1-polarizing or M2-polarizing conditions (figure 5H). LP-184–treated tumor cells promoted expression of MHC-I, MHC-IIA, and CD86 in LPS+IFN-γ–stimulated macrophages while suppressing the M2 marker CD206 under IL-4+IL-13 conditions (figure 5I,J), reflecting a phenotypic shift from tumor-supportive to immunostimulatory macrophages.

Finally, to evaluate whether this enhanced myeloid activation supports T-cell priming, LP-184–treated EO771-OVA tumor cells were co-cultured with BMDMs and naïve OT-I CD8^+^ T cells (figure 5K–M). LP-184 exposure markedly increased T-cell proliferation (figure 5L,M) and effector differentiation, evidenced by elevated IFN-γ, granzyme B, and perforin expression (figure 5M). Together, these results demonstrate that LP-184–treated tumor cells accumulate immunostimulatory ssDNA and acquire properties that activate DCs and macrophages, thereby linking innate immune sensing to adaptive CD8^+^ T-cell responses. Thus, pharmacologic induction of cytosolic ssDNA effectively recapitulates the immune-stimulatory effects of *TREX1* loss, establishing ssDNA accumulation as a central determinant of tumor immunogenicity.

### scRNA-seq reveals that LP-184 + anti-PD-1 remodels the tumor immune ecosystem by reducing suppressive macrophages and enhancing T-cell activation.

To better characterize the immune profile at molecular and single-cell levels, we performed scRNA-seq and flow cytometry on 4T1 tumors treated with vehicle (Ctrl), anti-PD-1, LP-184, or the combination after three doses of LP-184 and four doses of anti-PD-1 (online supplemental figure S4A). Consistent with figure 4, combination therapy significantly reduced tumor size and weight (online supplemental figure S4B and C), increased T-cell infiltration, and decreased M2-like macrophage infiltration (online supplemental figure S4D and E).

**Figure 6:**
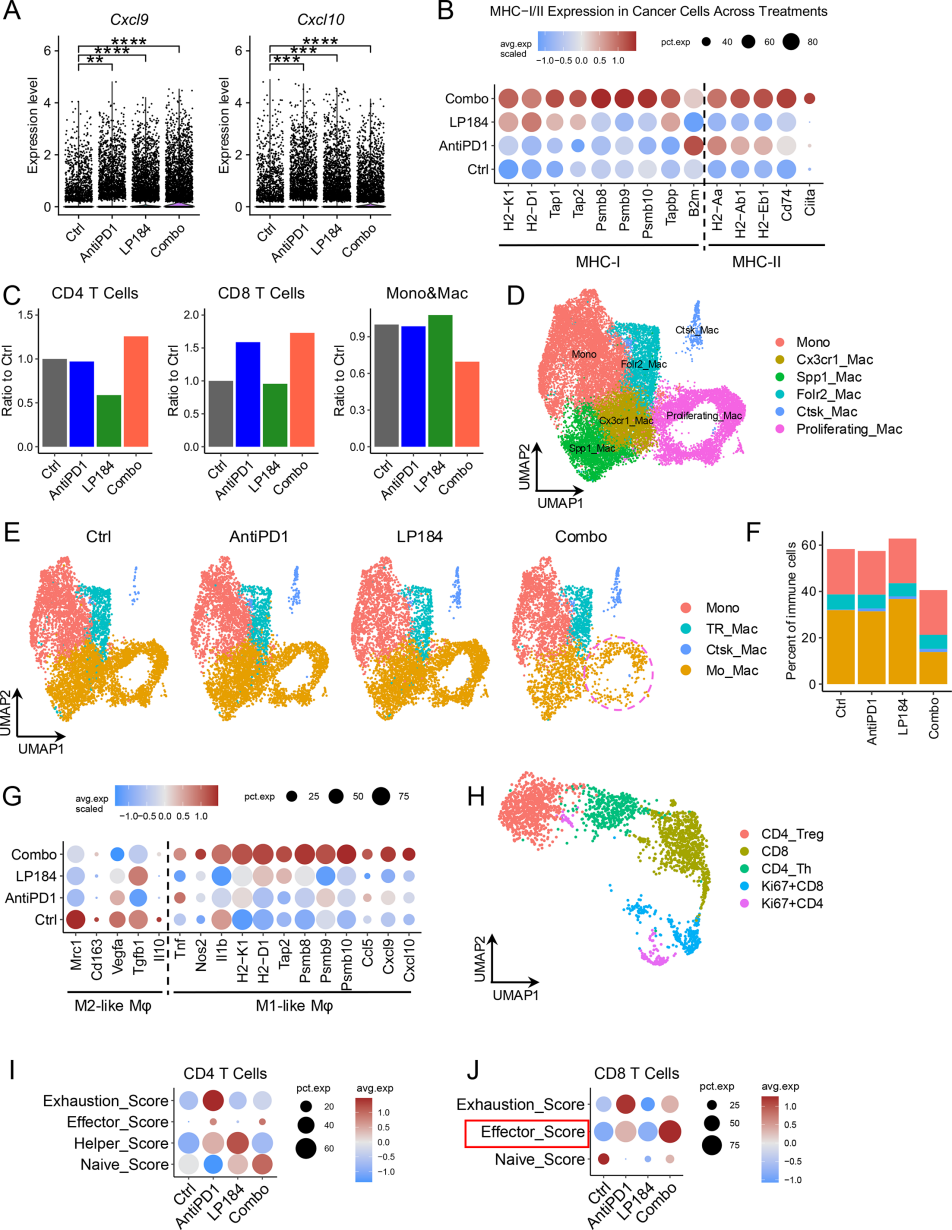
scRNA-seq reveals that LP-184+anti-PD-1 remodels the tumor immune ecosystem by reducing suppressive macrophages and enhancing T-cell activation. (**A**) Expression of *Cxcl9* and *Cxcl10* in tumor cells at single-cell level across treatment groups. (**B**) Heatmap showing MHC-I and MHC-II gene expression in tumor cells across treatments. (**C**) Relative abundance of CD4^+^ T cells, CD8^+^ T cells, and Mono&Mac populations normalized to control. (**D**) UMAP of macrophage clusters across treatment conditions. (**E**) Representative UMAPs of tumor-infiltrating immune cells across different treatment groups. Dashed circle indicates proliferating monocyte-derived macrophages. (**F**) Quantification of TR_Mac and Mo_Mac cluster composition across conditions. (**G**) Gene expression signature of M2- and M1-like macrophage markers in Mono_Mac. (**H**) UMAP visualization of T cell subsets. (I–J), functional module scoring in CD4 ^+^(**I**) and CD8 ^+^(**J**) T cells, computed with Seurat AddModuleScore (V.5.1.0) using the following gene sets: naive (*Ccr7, Sell, Lef1, Tcf7*), helper (*Ifng, Tnf, Il2, Il2ra, Il4, Il6, Il17a, Il21, Il22, Cd69, Cd74, (**H2–Aa***)), effector (*Prf1, Ifng, Nkg7, Gzmb, Gzma, Gzmk, Gzmd, Klrk1, Klrb1, Klrd1, Ctsw, Cst7*), and exhaustion (*Pdcd1, Tigit, Ctla4, Havcr2, Tox, Cd244a*). Scores reflect relative enrichment of each program per cell; data represent pooled tumors for each treatment group. TR_Mac, tissue-resident macrophage; Mo_Mac, monocyte-derived macrophage.

For scRNA-seq, tumor cells and immune subsets were partitioned using canonical markers (online supplemental figure S4F and G). IFN-related chemokines *Cxcl9* and *Cxcl10* were significantly upregulated in cancer cells with combination therapy (figure 6A). Downstream IFN programs showed enhanced antigen presentation: combination therapy increased both MHC-I and MHC-II, with LP-184 preferentially augmenting MHC-I and anti-PD-1 preferentially enhancing MHC-II, while the combination maximized both (figure 6B).

Immune composition analysis confirmed expansion of total T cells, including CD4^+^ and CD8^+^ T cell subsets, and contraction of monocyte and macrophages (Mono&Mac), most prominently with the combination (figure 6C–F**,** (online supplemental figure S4D and H). Within the Mono&Mac population, Uniform Manifold Approximation and Projection (UMAP) analysis identified Cx3cr1_Mac, Spp1_Mac, Folr2_Mac, Ctsk_Mac, and proliferating subsets (online supplemental figure S4I). Monocyte-derived macrophages (Mo_Mac), including Cx3cr1_Mac, Spp1_Mac, as well as a proliferating subset enriched for these populations, were markedly reduced by the combination treatment, with the most pronounced decrease observed in the proliferating subset, while tissue-resident Folr2_Mac (TR_Mac) was only minimally affected (figure 6D–F). To map CD206^+^ cells identified by flow cytometry, we examined Mrc1 expression across treatment groups and macrophage subclusters. Mrc1 was most highly expressed in the Folr2_Mac subset, with intermediate expression in Cx3cr1_Mac and Spp1_Mac populations, low expression in monocytes, and minimal to no expression in Ctsk_Mac. Overall, Mrc1 (CD206) expression decreased following combination treatment, consistent with the flow cytometry data (online supplemental figure S4J).

Marker analysis showed down-regulation of M2-associated genes (*Mrc1, Cd163, Vegfa, Tgfb1, Il10*) and induction of M1/immunostimulatory and antigen-processing genes (*Tnf, Nos2, H2-K1, H2-D1, Tap2, Psmb8, Psmb9, Psmb10, Ccl5, Cxcl9, Cxcl10*) in Mono&Mac cells (figure 6G). However, these M1-associated inflammatory and IFN-response genes were distributed across multiple macrophage subsets rather than defining a discrete M1 cluster (online supplemental figure S4K). Conventional dendritic cells displayed increased co-stimulatory and antigen-presentation programs (*Cd40, Cd86, H2-K1, H2-D1, Tap2, Psmb8, Psmb9, Psmb10, H2-Ab1, H2-Eb1, H2-DMb1*) plus T-cell-recruiting chemokines (*Cxcl9, Cxcl10*) and lymph-node homing receptors (*Ccr7, Ccr2*) under combination therapy (online supplemental figure S4M).

UMAP visualization of T cells revealed distinct subsets, including CD4^+^ regulatory T cells (CD4_Treg), CD4^+^ helper T cells (CD4_Th), CD8^+^ T cells, and proliferating Ki67^+^ CD4^+^ and CD8^+^ populations, and increased T cells are observed in combination treatment (figure 6H). Module scoring showed increased effector and helper signatures and reduced exhaustion in both CD4^+^ and CD8^+^ compartments, with the largest shifts in the combination group (figure 6I,J).

**Figure 7:**
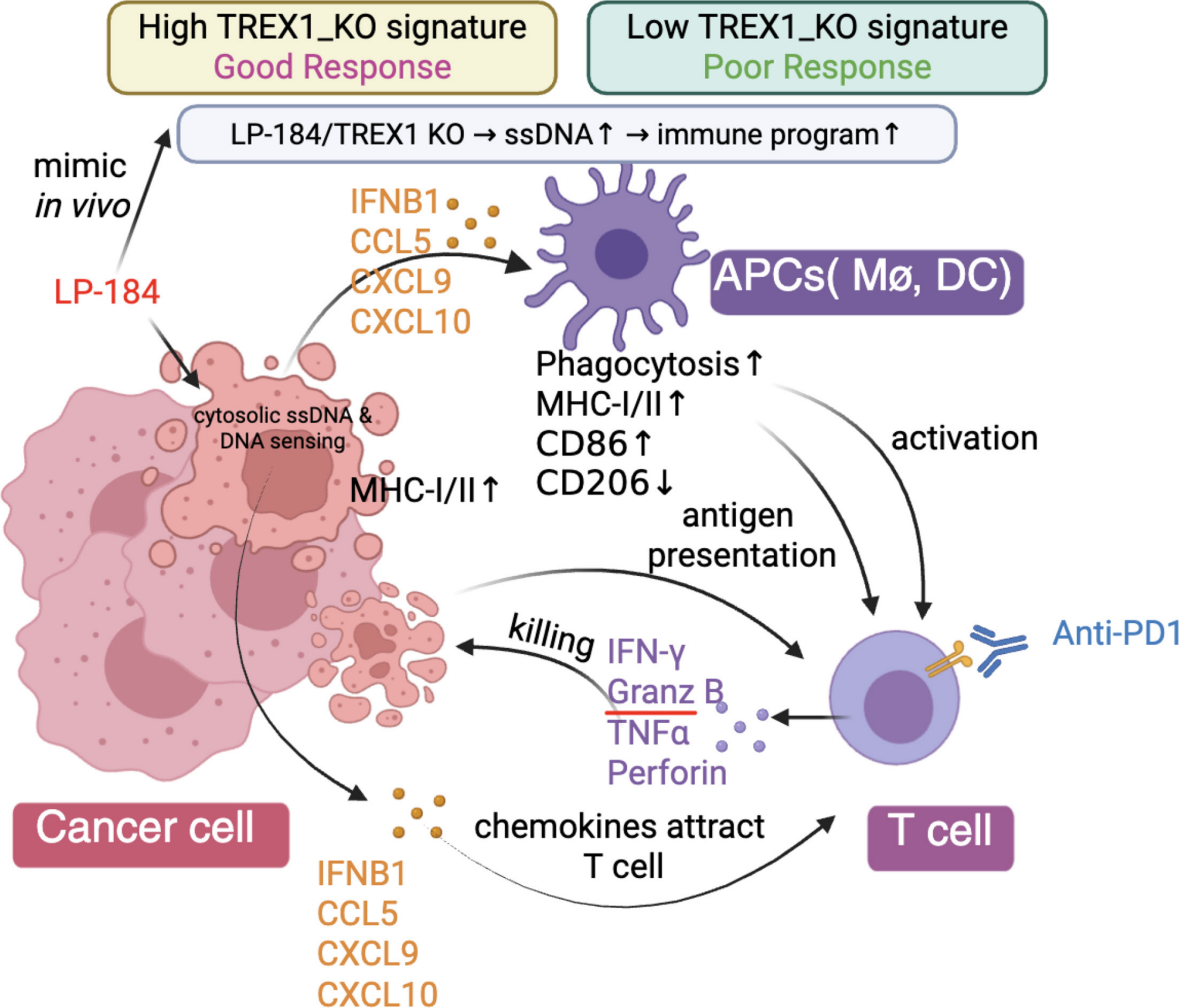
Mechanistic model showing how LP-184 mimics *TREX1* loss to induce cytosolic ssDNA, activate antitumor immunity, and enhance response to chemo-immunotherapy. LP-184 or *TREX1* deficiency increases cytosolic ssDNA in tumor cells, triggering DNA-sensing pathways and inducing type I interferons (eg, IFNB1) and chemokines (*CCL5*, *CXCL9*, *CXCL10*). These signals promote antigen presentation, phagocytosis, and activation of antigen-presenting cells (APCs), including macrophages and dendritic cells, leading to upregulation of MHC-I/II, increased CD86, and decreased CD206. The resulting inflammatory milieu enhances T-cell recruitment, activation, and cytotoxic function (IFN-γ, granzyme B, TNF-α, perforin), thereby improving antitumor immunity and sensitizing tumors to anti-PD-1 therapy. IFN, interferon; ssDNA, single-stranded DNA.

Together, these data show that LP-184 reshapes the TME by dampening immunosuppressive macrophage programs and enhancing tumor-cell antigen presentation and chemokine cues, thereby potentiating anti-PD-1 to drive robust effector T-cell infiltration and function (figure 7).

## Discussion

The absence of ER and HER2 targets in TNBC makes it one of the most difficult malignancies to treat. Conventional chemotherapies provide only transient benefit, and ICB induces durable responses in only a subset of patients.^1^ Previous studies, including ours^10^ and others,^11^,^15^ have shown that targeting the DNA exonuclease *TREX1* can enhance ICB efficacy by promoting cytosolic DNA accumulation and activating IFN-I signaling. Here, we extend this concept by demonstrating that the immune program induced by cytosolic ssDNA—rather than *TREX1* expression itself—is the key determinant of tumor immunogenicity and therapeutic responsiveness. This ssDNA-driven immunostimulatory signature serves as a robust biomarker that predicts chemo-immunotherapy response, providing a potential tool for clinical stratification.

By functionally screening NCCN-recommended chemotherapeutics and other agents in development, we identified LP-184—an acylfulvene-derived alkylating agent in clinical development—as a potent pharmacologic inducer of cytosolic ssDNA that phenocopies *TREX1* deficiency. LP-184 retains the cytotoxic activity typical of chemotherapy while broadening immune responsiveness through enhanced antigen presentation, chemokine production, and T-cell infiltration. Mechanistically, LP-184–induced ssDNA accumulation likely arises from DNA damage-associated replication stress and repair intermediates, thereby enhancing ssDNA generation upstream, in contrast to TREX1 ablation, which increases cytosolic DNA primarily through impaired degradation. From a translational perspective, ssDNA accumulation defines a convergent and actionable axis linking genomic instability with immune activation. Measuring ssDNA levels or ssDNA-induced transcriptional signatures could improve patient stratification for chemo-immunotherapy combinations. Moreover, LP-184 exemplifies a new drug development paradigm—designing or engineering chemotherapeutic agents that preferentially generate cytosolic ssDNA. This strategy bridges classical chemotherapy with modern immunotherapy and provides a rational framework to expand the population of patients who benefit from ICB.

While paclitaxel and doxorubicin also induced ssDNA accumulation, their effects plateaued at higher doses, whereas LP-184 elicited a more sustained dose-dependent increase. Notably, LP-184-induced ssDNA accumulation was not accompanied by substantial ATP or HMGB1 release (data not shown), suggesting a mode of immune activation less reliant on classical DAMP-driven inflammation and more on nucleic acid-associated sensing. Consistent with this, scRNA-seq analysis showed that LP-184 reduced immunosuppressive monocyte-derived macrophage populations while enhancing T-cell effector function, indicating a more favorable immune landscape. Enrichment of TNFα signaling via NFκB observed in LP-184-treated tumors suggests engagement of inflammatory pathways downstream of DNA damage and nucleic acid sensing. Given the context-dependent roles of NFκB signaling in tumor biology, this pathway may contribute to shaping the tumor immune microenvironment. Together, these findings suggest that LP-184 differs from conventional chemotherapies not only in the magnitude but also in the quality of immune activation and may be particularly effective in tumors with suppressive myeloid microenvironments.

Beyond pharmacologic mimicry of *TREX1* loss, our findings suggest the potential to develop dual-function agents that both induce ssDNA production and prevent its degradation by inhibiting TREX1 enzymatic activity. Such compounds could sustain cytosolic ssDNA signaling even in tumors with low basal ssDNA levels, thereby overcoming a key limitation of single-function TREX1 inhibitors. This strategy would enable persistent innate immune activation without compromising DNA repair homeostasis, providing a mechanistically grounded framework for rational drug design.

Mechanistically, our findings distinguish the immune consequences of ssDNA versus dsDNA accumulation. While cytosolic dsDNA potently activates the cGAS–STING pathway and often reflects severe genotoxic stress leading to cell death,^7^ ssDNA exhibits distinct immunogenic behavior.^9 27^ It can persist in viable tumor cells, sustaining lower-grade IFN signaling that chronically promotes immune recruitment. Thus, ssDNA functions as a tunable and therapeutically tractable immunogenic intermediate that bridges DNA damage to adaptive immune activation under controlled pharmacologic modulation.

Together, these insights delineate a coherent mechanistic and translational model in which tumor-intrinsic ssDNA generation serves as the molecular bridge between DNA damage and antitumor immunity, providing both a biomarker for therapy selection and a foundation for developing ssDNA-based chemo-immunotherapy strategies in TNBC and beyond.

## Methods

### Cell lines and reagents

TNBC cell lines (MDA-MB-231, BT-549, CAL-51, 4T1, T11, and EO771-OVA) were maintained at 37 °C in a humidified incubator (5% CO₂). Cell lines were obtained from ATCC, DSMZ, CH3 Biosystems, or the Characterized Cell Line Core Facility at The University of Texas MD Anderson Cancer Center. Primary antibodies were: TBK1 (Cell Signaling Technology (CST), #3504), phospho-TBK1 (Ser172; CST, #5483), IRF3 (CST, #11904), phospho-IRF3 (Ser396; CST, #29047), STING (mouse; CST, #16 029T), TREX1 (mouse; CST, #611987), and TREX1 (human; Santa Cruz Biotechnology, sc-271870). The antibodies used for flow cytometry were purchased from BioLegend or BD Biosciences. Zombie Aqua Fixable Viability Dye (423101) and Cell Activation Cocktail (with Brefeldin A, 423304) were obtained from BioLegend. The following mouse antibodies were from BioLegend: CD3 (PerCP-Cy5.5 100218, BV785 100232), CD11b (BV785 101243), CD11c (Pacific Blue 117322), CD16/32 (101302), CD206 (AF647 141712), PD-1 (PE-Cy7 135216), PD-L1 (PE 124308, BV605 153606), EpCAM (BV650 118241), Ly6C (PE-Cy7 128018), Ly6G (PerCP 127654), F4/80 (FITC 157310), IFN-γ (AF700 505824), TNF-α (AF488 506313), perforin (Pacific Blue 154408), granzyme B (PE 372208), CD45.2 (BV421 109832, APC-Cy7 109824), MHC-IIA (APC-Cy7 107628), and PDGFR-α (PE/Dazzle 594 135922). The following antibodies were from BD Biosciences: TCRβ (BUV395 569248), CD4 (BUV661 612974), CD8 (BUV805 612898), and CD103 (PE 566844). Small-molecule agents were sourced from Selleckchem or MedChemExpress; LP-184 was a gift from Lantern Pharma.

## Supplementary material

10.1136/jitc-2025-014722 online supplemental file 1

## Data Availability

Data are available in a public, open access repository. Data are available upon reasonable request.
