# Supplementary material for: Chemotherapeutic induction of cytosolic single-stranded DNA accumulation sensitizes triple-negative breast cancer to immunotherapy
Source: J Immunother Cancer. 2026 Jun 29;14(6):e014722. doi: 10.1136/jitc-2025-014722 (PMC13331146; doi:10.1136/jitc-2025-014722)
Supplement: online supplemental file 1 [file jitc-14-6-s001.pdf]

Supplemental Materials

This section includes Supplemental Methods and Supplemental Figures S1–S4.

Supplemental Methods

Chemotherapeutic drug screening

For the ssDNA-induction screen, the chemotherapy panel comprised alkylating agents (dacarbazine, carmustine, melphalan, busulfan, cyclophosphamide, chlorambucil); platinum compounds (cisplatin, carboplatin, oxaliplatin); antimetabolites (5-fluorouracil, gemcitabine, methotrexate, pemetrexed); topoisomerase inhibitors (irinotecan, topotecan, doxorubicin, etoposide); and others (mitomycin C, bleomycin, hydroxyurea, and the acylfulvene analog LP-184). DMSO served as the vehicle control, and hydroxyurea served as the positive control. Unless noted, each drug was tested at 10 µM and 2 µM for 48h; hydroxyurea was tested at 10 mM and 2 mM. All conditions were performed in three technical replicates using MDA-MB-231 cells. Intracellular ssDNA accumulation was quantified using a flow cytometry–based assay following drug treatment. Detailed procedures are described in the "Flow cytometry—Cytosolic ssDNA staining" section of the Methods. Key findings were subsequently validated across multiple independent cell lines.

Tumor models

Six- to eight-week-old female BALB/c mice were purchased from Charles River. All animal experiments were approved by the Institutional Animal Care and Use Committee (IACUC). Murine mammary carcinoma cells (T11 or 4T1; 5 × 10^4 cells per mouse) were orthotopically implanted into the mammary fat pad. Tumors were allowed to reach 4–5 mm in diameter (7~8

days post-implantation) before randomization and treatment initiation. Tumor length (L) and width (W) were measured by calipers, and volume was calculated as $V=(L\times W^2)/2$. Treatments were administered for up to 1 month; remaining mice were subsequently monitored for long-term survival. For immune checkpoint blockade, mice received intraperitoneal (i.p.) injections three times per week (days 1/3/5) of 200 µg anti-PD-1 (rat IgG2a, clone RMP1-14) or isotype controls (rat IgG2a, clone 2A3; polyclonal hamster IgG, cat. #BE0087; all from Bio X Cell). LP-184 (3.5 mg/kg) was administered i.p. every 3 days for a total of three doses. ssDNA (3.75 mg/kg), formulated with liposomes, was intravenously given twice weekly (days 1 and 5 each week) for up to 1 month.

TREX1_KO signature generation and patient data analysis

To derive transcriptional signatures representing *TREX1* deficiency, RNA sequencing was performed on CRISPR/Cas9-engineered CAL51 *TREX1*-knockout (KO) cells (vs control) and on *in vivo* 4T1 *Trex1*-KO tumors treated with control IgG or anti–PD-1 for 1 week[1]. Differential expression was computed with edgeR (v4.2.0) using $|\log_2 \text{fold change}| > 0.5$ and $FDR < 0.1$ unless otherwise specified. Mouse genes from the 4T1 datasets were converted to their human orthologs prior to signature construction. Each comparison (CAL51 KO vs Ctrl; 4T1 KO+IgG vs WT+IgG; 4T1 KO+ICB vs WT+ IgG) generated a context-specific set of DEGs, which was used to define a corresponding *TREX1_KO* signature, with gene weights equal to their $\log_2$FC. For each sample, the signature score was computed as a weighted sum of row-wise z-scored $\log_2$(TPM+1) expression, using the formula: $\text{Score} = \Sigma(w\_g \times z\_g) / \Sigma|w\_g|$, where w_g is the $\log_2$FC-based weight for gene g and z_g is the per-gene z-score. Unless otherwise specified, the *in vivo* 4T1 Trex1 KO+IgG vs WT+IgG–derived signature was used for downstream patient analyses. Patient transcriptomic datasets included TONIC trial[2], GSE241876[3],

GSE194040[4]. Only baseline biopsies from patients receiving chemo-immunotherapy were analyzed. Associations between *TREX1*_KO scores and outcome used two-sided t-tests (normal data) or Wilcoxon rank-sum tests (non-normal). Predictive performance was evaluated by ROC/AUC (R pROC). Benchmarks included PD-L1 expression, TMB, the IFN-γ/T-cell–inflamed signature, and the cytolytic activity score[5–9]. Pathway enrichment used clusterProfiler (v4.10.0) with MSigDB Hallmark (v7.5.1) gene sets as previously described[10].

Western blot

Cells were lysed in urea buffer (8 M urea, 150 mM β-mercaptoethanol, 50 mM Tris-HCl, pH 7.5) by sonication and clarified by centrifugation. Protein concentration was measured using the Bradford assay (Bio-Rad). Equal amounts of protein were resolved by SDS–PAGE, transferred to nitrocellulose membranes (Bio-Rad), and probed with the indicated antibodies.

qRT-PCR

Total RNA was isolated from cultured cells using the QIAGEN RNeasy Plus Kit. Isolated RNA (250 ng) was reverse transcribed to cDNA with the iScript cDNA Synthesis Kit (Bio-Rad). Quantitative PCR was performed on a Bio-Rad CFX instrument using iQ SYBR Green Supermix (Bio-Rad). Gene expression was normalized to UBI. Primer sequences were as follows: Mouse *Ubi*: Forward: TGGCTATTAATTATTCGGTCTGCAT; Reverse: GCAAGTGGCTAGAGTGCAGAGTAA; Mouse *Ifnb1*: Forward: CAGCTCCAAGAAAGGACGAAC; Reverse: GGCAGTGTAACTCTTCTGCAT; Mouse *Cxcl10*: Forward: GCCGTCATTTTCTGCCTCAT; Reverse: GCTTCCCTATGGCCCTCATT; Mouse *Ccl5*: Forward: TGCCCACGTCAAGGAGTATTTC; Reverse: AACCCACTTCTTCTCTGGGTTG

ssDNA–liposome formulation and *in vivo* administration

Three synthetic ssDNA oligonucleotides (5′-AAACGTGGGTCATCTTTGGCATAACGG-3′; 5′-CCACTCCTGATCACCCCCCGCCGACAAAA-3′; 5′-GGGTCAAAGAAGACATGTCAAGAGAAATTTAAAAAT-3′) were described previously[1] and mixed at a 1:1:1 mass ratio, dissolved in nuclease-free water to 1 mg/mL, and stored at the recommended temperature until use. The protocol for preparation and administration was adapted from previously published methods[11,12], and is briefly described as follows: Cationic liposomes were prepared by dissolving 100 mg DOTMA (1,2-di-O-octadecenyl-3-trimethylammonium propane) and 40.92 mg DOPE (dioleoylphosphatidylethanolamine) in 680 μL absolute ethanol. The lipid mixture was added dropwise into 34 mL double-distilled water under constant stirring (200 rpm, 25 °C) for 1 h. The suspension was filtered through a 0.45 μm syringe filter to remove aggregates and stored at 4 °C. For each mouse, 75 μg ssDNA was mixed with 25 μL Opti-MEM, then combined with 75 μL liposome solution pre-diluted in 25 μL Opti-MEM. The mixture was incubated at room temperature for 20 min before injection.

Immunostaining of ssDNA and microscopy

The cytosolic ssDNA was stained essentially as described[13]. 231 cells grown on glass coverslips were treated with the indicated drugs at 0.4 μM for 48 h, fixed in 2% paraformaldehyde in PBS for 15 min at room temperature, and washed three times with PBS. Cells were then permeabilized with 0.5% saponin in PBS for 10 min to allow cytoplasmic access without disrupting nuclear or mitochondrial membranes. After permeabilization, samples were blocked with 5% horse serum in PBS for 30 min, followed by incubation with mouse anti-ssDNA antibody (Millipore, MAB3868; 50 μg/mL) diluted in permeabilization buffer for 1 h at

room temperature. Approximately 20 μL of antibody solution was used per 18 mm coverslip, and incubation was performed in a humidified chamber with coverslips inverted onto antibody droplets placed on Parafilm. For S1 nuclease treatment, samples were incubated with S1 nuclease after saponin permeabilization and before ssDNA antibody incubation, following the manufacturer's instructions. After primary antibody incubation, coverslips were washed five times with PBS for a total of 30 min, then incubated with Alexa Fluor 488–conjugated anti-mouse secondary antibody (1:500 dilution) for 30 min at room temperature. Samples were washed again five times in PBS for at least 30 min, mounted with antifade reagent containing DAPI (Invitrogen), and imaged using a Nikon fluorescence microscope with a 60× objective lens.

Single-cell RNA sequencing (scRNA-seq)

50,000 4T1 cells were orthotopically implanted into the mammary fat pad of female BALB/c mice on day 0 (D0). On day 7, when tumors reached 4–5 mm in diameter, mice were randomized into four treatment groups: vehicle control, anti–PD-1 (10 mg/kg, i.p.), LP-184 (3.5 mg/kg, i.p.), or the combination. Anti–PD-1 was administered on days 7, 10, 13, and 15, while LP-184 was administered on days 7, 10, and 13. On day 16, tumors were excised and dissociated into single-cell suspensions by enzymatic digestion with 0.25 mg/ml Liberase TL (Roche, 05401020001) and 0.1 mg/ml DNase I (Roche, 11284932001) for 60 min at 37 °C with rotation, followed by pass through a 70 μm strainer and red blood cell lysis. Single-cell suspensions were split for flow cytometry analysis or cryopreserved until use for scRNA-seq. For scRNA-seq, cells were thawed, and live cells were enriched by DAPI-negative flow cytometry sorting. For each treatment group, equal numbers of cells from individual mice were pooled (4 tumors for Ctrl,

anti–PD-1, and LP-184; 5 tumors for combination). Approximately 20,000 viable cells per group were targeted for recovery and loaded onto the 10x Genomics Chromium Controller following the manufacturer's protocol.

Single-cell cDNA libraries were generated using the GEM X Single cell 3' Gem kit v4 (10x Genomics) and sequenced on an Illumina NovaSeq X platform to an average depth of ~30,000 reads per cell. Raw sequencing data were processed using Cell Ranger (10x Genomics) against the mm10 reference genome to generate gene–barcode matrices. Downstream analyses, including quality control, normalization, clustering, and visualization, were performed in Seurat (v5.1.0, R) similarly as previously described[14]. Cells with <200 detected genes or >20% mitochondrial transcripts were excluded. Putative doublets were removed by excluding cells co-expressing markers of multiple lineages.

Flow cytometry

Cytosolic ssDNA staining. Adherent cultured cells were treated with LP-184 with indicated concentration and time. Cells were detached with TrypLE, neutralized with 275 µL complete medium, transferred to a 96-well round-bottom plate, and centrifuged 600 g, 3 min between steps. Cells were washed with 200 µL PBS and stained with dead/live dye Zombie Aqua (50 µL, 1:500, 10 min, RT, dark). After blocking, cells were fixed in 100 µL 2% PFA (15 min, RT). Cell membrane (but not nuclear membrane) was permeabilized with PBS/0.1% saponin/1% BSA, washed with permeabilized buffer twice, and stained for ssDNA with anti-ssDNA (MAB3868, 20 µg/mL; 1:50 in permeabilization buffer; 60 min, RT); an AF647/AF488 labeled anti-mouse (1:400, 30 min, RT, dark) was used for secondary antibody staining. Samples were washed

twice, resuspended in FACS buffer, and acquired within a few hours. Data were gated as singlets and live cells and reported as MFIs.

Staining of tumor-infiltrating immune cells. Tumors were excised and dissociated to single-cell suspensions by enzymatic digestion with Liberase TL (0.25 mg/mL; Roche 05401020001) and DNase I (0.1 mg/mL; Roche 11284932001) for 60 min at 37 °C with rotation, filtered through a 70 μm strainer, and subjected to RBC lysis. Cells were split for flow cytometry. For surface immunophenotyping, cells were stained with Zombie Aqua viability dye (1:250, 10 min, RT), Fc-blocked with anti-CD16/32 (1:100, 10 min, 4 °C), and incubated with an antibody cocktail against surface immune markers for 30 min at 4 °C; samples were washed and acquired within a few hours. For T-cell cytotoxic readouts, cells were stimulated with Cell Activation Cocktail with Brefeldin A (BioLegend 423303) for 4 h at 37 °C, stained for surface markers as above, fixed and permeabilized using the Foxp3/Transcription Factor Staining Buffer Set (Invitrogen 00-5523-00), and then stained in permeabilization buffer for intracellular IFN-γ, TNF-α, granzyme B, and perforin for 60 min at RT; samples were washed twice and acquired the same day. After exclusion of dead cells and doublets, T cells were gated as $CD45^{+}CD11b^{-}CD3^{+}$ (or $TCR\beta^{+}$), with $CD4^{+}$ and $CD8^{+}$ subsets defined by CD4 and CD8 expression; M2-like macrophages were defined as $CD45^{+}CD11b^{+}Ly6C^{-}Ly6G^{-}CD3^{-}F4/80^{+}CD206^{+}$; FN-γ, TNF-α, granzyme B, and perforin positive cells were gated on CD8 T cells (**Supplementary Fig. S3A-B**).

Generation of knockout cell lines

Human *TREX1* gRNA sequences (#1: GAGAGCTTGTCTACCACACG; #2: GAGCATCCACCCACCGCAGC) were cloned into the lentiCRISPR_v2 plasmid, and lentivirus

was produced to transduce CAL-51 cells. Puromycin selection was applied to establish CAL-51 *TREX1* knockout cell lines. To generate 231 *TREX1* knockout cells, gRNA sequences (#1: TCTGGATGGTGCCTTCTGTG; #2: GAGAGCTTGTCTACCACACG) were synthesized by Synthego. Electroporation was performed with 90 pmol sgRNA complexed with 20 pmol Cas9 protein in 100,000 cells, following the manufacturer's protocol (Invitrogen, Lonza system) using a single pulse at 1700 V in a 0.1 µm cuvette. Cells were subsequently cultured, and *TREX1* knockout was verified by Western blot.

Co-culture of cancer cell and immune cells

Phagocytosis assay. 4T1 tumor cells were treated with LP-184 (10 µg/mL) or DMSO for 48 h. Cells were detached using TrypLE + 2 mM EDTA and labeled with CellTrace™ Far Red (CTFR; Thermo Fisher) according to the manufacturer's instructions. Labeled tumor cells (4×10^4 cells) were co-cultured with BMDCs (differentiated with GM-CSF + IL-4 for 7 days) or BMDMs (differentiated with M-CSF for 7 days) at a 1 : 2.5 tumor-to-immune-cell ratio (1×10^5 immune cells per well) in 96-well round-bottom plates. After 4 h of co-culture at 37 °C, cells were harvested, stained for EpCAM-BV650 (4T1), CD11c (BMDCs), or F4/80 (BMDMs), and analyzed by flow cytometry. Phagocytosis was quantified as CTFR mean fluorescence intensity (MFI) within gated $CD11c^+$ or $F4/80^+$ populations (excluding $EpCAM^+$ tumor cells), normalized to vehicle controls. **Macrophage polarization assay.** LP-184–treated 4T1 tumor cells (10 µM, 48 h) were detached and co-cultured with BMDMs at a 1 : 2.5 ratio ($4 \times 10^4 : 1 \times 10^5$ cells) in 96-well flat-bottom plates. Immediately upon co-culture, macrophages were stimulated with either M1-polarizing medium (LPS 100 ng/mL + IFN-γ 20 ng/mL, 24 h) or M2-polarizing medium (IL-4 20 ng/mL + IL-13 20 ng/mL, 24 h). Cells were stained for MHC-I, MHC-II,

CD86 (M1 markers), and CD206 (M2 marker). Flow-cytometric MFIs were normalized to BMDMs cultured without tumor cells (set to 1.0). **T-cell priming assay.** EO771-OVA tumor cells were treated with LP-184 (10 µg/mL, 48 h) or vehicle, detached, and co-cultured with BMDMs (4×10^4: 1×10^5 cells; 1 : 5 ratio) for 4 h to allow antigen uptake and presentation. CTFR-labeled naïve OT-I $CD8^+$ T cells (2×10^5 cells) were then added and co-cultured for an additional 3 days. Cells were analyzed by flow cytometry for proliferation (CTFR dilution) and activation markers IFN-γ, Granzyme B, and Perforin. Percentages of CTFR-low proliferative and cytokine-positive OT-I T cells were quantified within the $CD8^+$ gate.

Bulk RNAseq sequencing and analysis

CAL-51 *TREX1* knockout cells and 4T1 cells treated with LP-184 for 48 h were harvested for RNA extraction using the RNeasy Plus kit (QIAGEN). For each condition, three independent biological replicates were prepared. RNA samples were submitted to Novogene for library construction and sequencing. Raw reads were processed as previously described[10]. Differential expression analysis was performed in R using edgeR, and P values were adjusted using the Benjamini–Hochberg procedure to control the false discovery rate (FDR).

Statistical analysis

Statistical analyses were performed using R and GraphPad Prism. Unless otherwise specified, two-group comparisons were assessed by two-sided Student's *t*-test; for non-normally distributed data, the Wilcoxon rank-sum test (unpaired) or Wilcoxon signed-rank test (paired) was applied. Comparisons among more than two groups were evaluated by two-way ANOVA. For tumor

growth curves, two-way ANOVA was used to assess group differences at the endpoint. Multiple comparisons were corrected using the Benjamini–Hochberg false discovery rate (FDR) method.

Supplemental references

1 Zhang J, Dai H, Huo L, *et al.* Cytosolic DNA accumulation promotes breast cancer immunogenicity via a STING-independent pathway. *J Immunother Cancer*. 2023;11. doi: 10.1136/JITC-2023-007560

2 Voorwerk L, Slagter M, Horlings HM, *et al.* Immune induction strategies in metastatic triple-negative breast cancer to enhance the sensitivity to PD-1 blockade: the TONIC trial. *Nature Medicine 2019 25:6*. 2019;25:920–8. doi: 10.1038/s41591-019-0432-4

3 Wilkerson AD, Parthasarathy PB, Stabellini N, *et al.* Phase II Clinical Trial of Pembrolizumab and Chemotherapy Reveals Distinct Transcriptomic Profiles by Radiologic Response in Metastatic Triple-Negative Breast Cancer. *Clin Cancer Res*. 2024;30:82–93. doi: 10.1158/1078-0432.CCR-23-1349

4 Wolf DM, Yau C, Wulfkuhle J, *et al.* Redefining breast cancer subtypes to guide treatment prioritization and maximize response: Predictive biomarkers across 10 cancer therapies. *Cancer Cell*. 2022;40:609-623.e6. doi: 10.1016/j.ccell.2022.05.005

5 Schmid P, Adams S, Rugo HS, *et al.* Atezolizumab and Nab-Paclitaxel in Advanced Triple-Negative Breast Cancer. *New England Journal of Medicine*. 2018;379:2108–21. doi: 10.1056/NEJMOA1809615;ISSUE:ISSUE:DOI

6 Ayers M, Lunceford J, Nebozhyn M, *et al.* IFN-γ–related mRNA profile predicts clinical response to PD-1 blockade. *J Clin Invest*. 2017;127:2930–40. doi: 10.1172/JCI91190

7 Cristescu R, Mogg R, Ayers M, *et al.* Pan-tumor genomic biomarkers for PD-1 checkpoint blockade-based immunotherapy. *Science (1979)*. 2018;362. doi: 10.1126/SCIENCE.AAR3593;WEBSITE:WEBSITE:AAAS-SITE;JOURNAL:JOURNAL:SCIENCE;WGROUP:STRING:PUBLICATION

8 Rizvi NA, Hellmann MD, Snyder A, *et al.* Mutational landscape determines sensitivity to PD-1 blockade in non–small cell lung cancer. *Science*. 2015;348:124. doi: 10.1126/SCIENCE.AAA1348

9 Rooney MS, Shukla SA, Wu CJ, *et al.* Molecular and genetic properties of tumors associated with local immune cytolytic activity. *Cell*. 2015;160:48–61. doi: 10.1016/j.cell.2014.12.033

10 Du Y, Ah Kioon MD, Laurent P, *et al.* Chemokines form nanoparticles with DNA and can superinduce TLR-driven immune inflammation. *Journal of Experimental Medicine*. 2022;219. doi: 10.1084/JEM.20212142/213252

11 Zhao GX, Bu GL, Liu GF, *et al.* mRNA-based Vaccines Targeting the T-cell Epitope-rich Domain of Epstein Barr Virus Latent Proteins Elicit Robust Anti-Tumor Immunity in Mice. *Advanced Science*. 2023;10:2302116. doi: 10.1002/ADVS.202302116;SUBPAGE:STRING:FULL

12 Kranz LM, Diken M, Haas H, *et al.* Systemic RNA delivery to dendritic cells exploits antiviral defence for cancer immunotherapy. *Nature 2016 534:7607*. 2016;534:396–401. doi: 10.1038/nature18300

13 McGrail DJ, Pilié PG, Dai H, *et al.* Replication stress response defects are associated with response to immune checkpoint blockade in nonhypermutated cancers. *Sci Transl Med*. 2021;13:6201. doi: 10.1126/SCITRANSLMED.ABE6201;ISSUE:ISSUE:DOI

14 Du Y, Faz-Lopez B, Ah Kioon MD, *et al.* Altered X-chromosome inactivation of the TLR7/8 locus and heterogeneity of pDCs in systemic sclerosis. *J Exp Med*. 2025;222. doi: 10.1084/JEM.20231809

Supplementary Fig.1: An *in vivo* *TREX1*-deficiency signature, rather than *TREX1* expression, predicts chemo-immunotherapy response in TNBC.

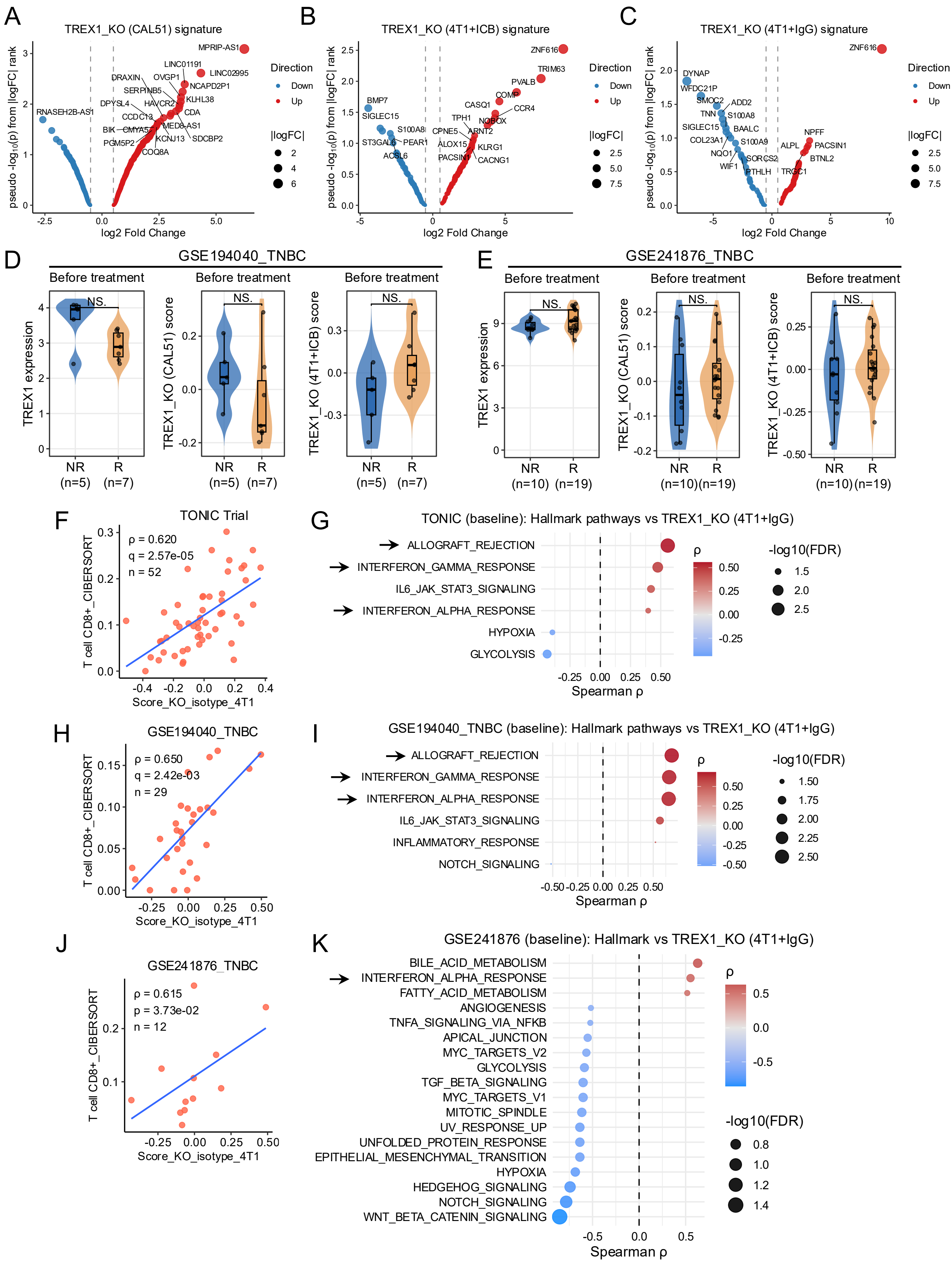

Supplementary Fig.1: An *in vivo TREX1*-deficiency signature, rather than *TREX1* expression, predicts chemo-immunotherapy response in TNBC.

A–C, Volcano plots showing differential expression from RNA-seq of CAL51 (**A**, *TREX1*-KO vs Ctrl), 4T1 + ICB (**B**, *TREX1*-KO + anti-PD1 vs Ctrl), and 4T1 + IgG (**C**, *TREX1*-KO + IgG vs Ctrl) tumors. Genes meeting thresholds ($|\log_2 FC| > 0.5$, FDR < 0.1) are highlighted in red (up) and blue (down). The top 20 genes with the largest $|\log_2 FC|$ values are labeled. The y-axis represents the pseudo $\log_{10}|\text{logFC}|$-rank. **D–E,** Violin/box overlays comparing *TREX1* expression and *TREX1*_KO signature scores between non-responders (NR) and responders (R) at pre-treatment in GSE194040 (**D**) and GSE241876 (**E**) TNBC datasets. Central dot indicates the median; *P* values were determined by two-sided Wilcoxon rank-sum test. **F–K,** Correlations between the *TREX1*_KO (4T1 + IgG) score and $CD8^+$ T-cell abundance (CIBERSORT) in the TONIC trial (**F**) , GSE194040 (**H**) and GSE241876 (**J**). Spearman ρ and adjusted *P* values are shown. **G, I, K,** Dot plots showing Spearman correlations between Hallmark ssGSEA pathways and the *TREX1*_KO (4T1 + IgG) score in baseline samples from TONIC (**G**), GSE194040 (**I**) and GSE241876 (**K**). Color encodes ρ and point size encodes $-\log_{10}$(FDR); only pathways meeting $|\rho| > 0.2$ are labeled.

Supplementary Fig.2: LP-184 and ssDNA enhance anti–PD-1 efficacy by increasing CD8^{+} T-cell cytotoxic function and reducing M2 macrophages *in vivo*.

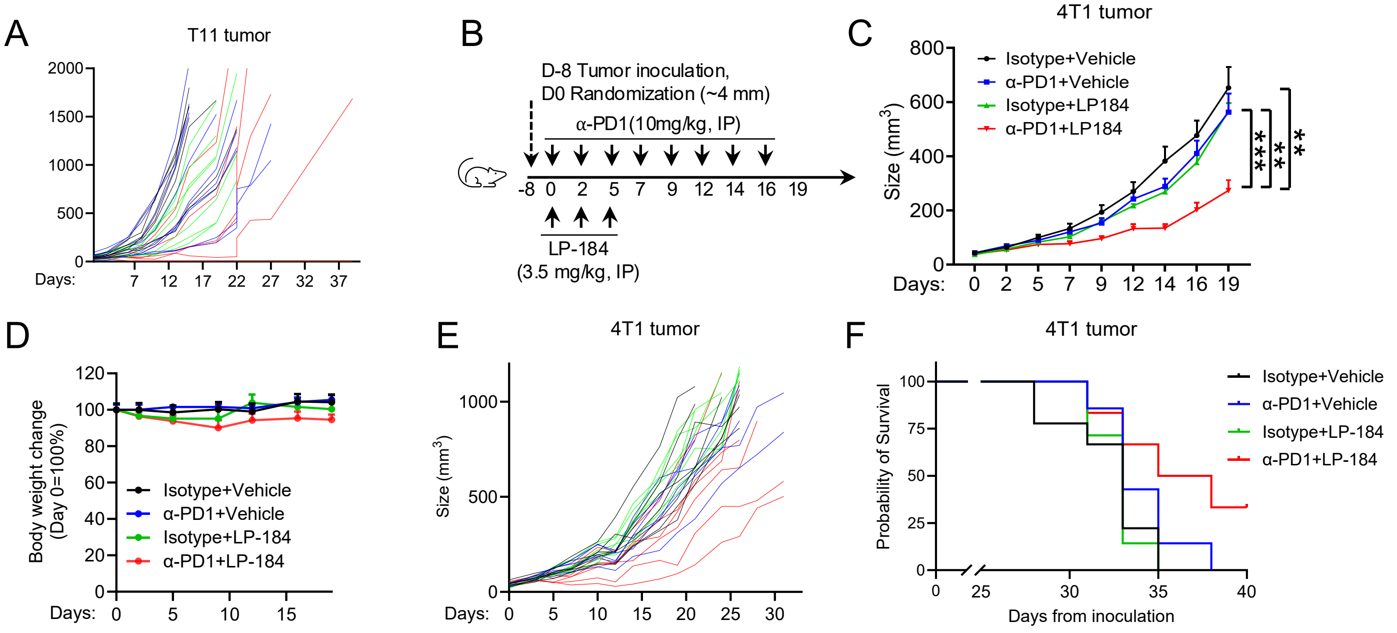

A, Individual tumor growth trajectories of T11 tumors under indicated treatments: Isotype + Vehicle (n = 7), α–PD-1 + Vehicle (n = 9), Isotype + LP-184 (n = 6), and α–PD-1 + LP-184 (n = 6). **B–D,** Experimental design for 4T1 (**B**), tumor growth curves (**C**; Isotype + Vehicle (n = 10), α–PD-1 + Vehicle (n = 10), Isotype + LP-184 (n = 6), α–PD-1 + LP-184 (n = 7)), and body weight changes (**D**) of 4T1 models under indicated treatments. **E–F,** Individual tumor growth trajectories of 4T1 (**E**; Isotype + Vehicle (n = 7), α–PD-1 + Vehicle (n = 7), Isotype + LP-184 (n = 7), α–PD-1 + LP-184 (n = 6)) and Kaplan–Meier survival analysis of 4T1 tumors (**F**; Isotype + Vehicle (n = 7), α–PD-1 + Vehicle (n = 7), Isotype + LP-184 (n = 7), α–PD-1 + LP-184 (n = 6)) under indicated treatments. Data are mean ± s.e.m.; significance symbols: $P < 0.05$, $*P < 0.01$, $**P < 0.001$. Statistical analysis: tumor growth analyzed by repeated-measures two-way ANOVA with Dunnett's multiple comparisons; survival analyzed by Kaplan–Meier and log-rank test.

Supplementary Fig.3: Representative flow-cytometric gating strategy for immune cells, cytotoxic molecules and phagocytosis.

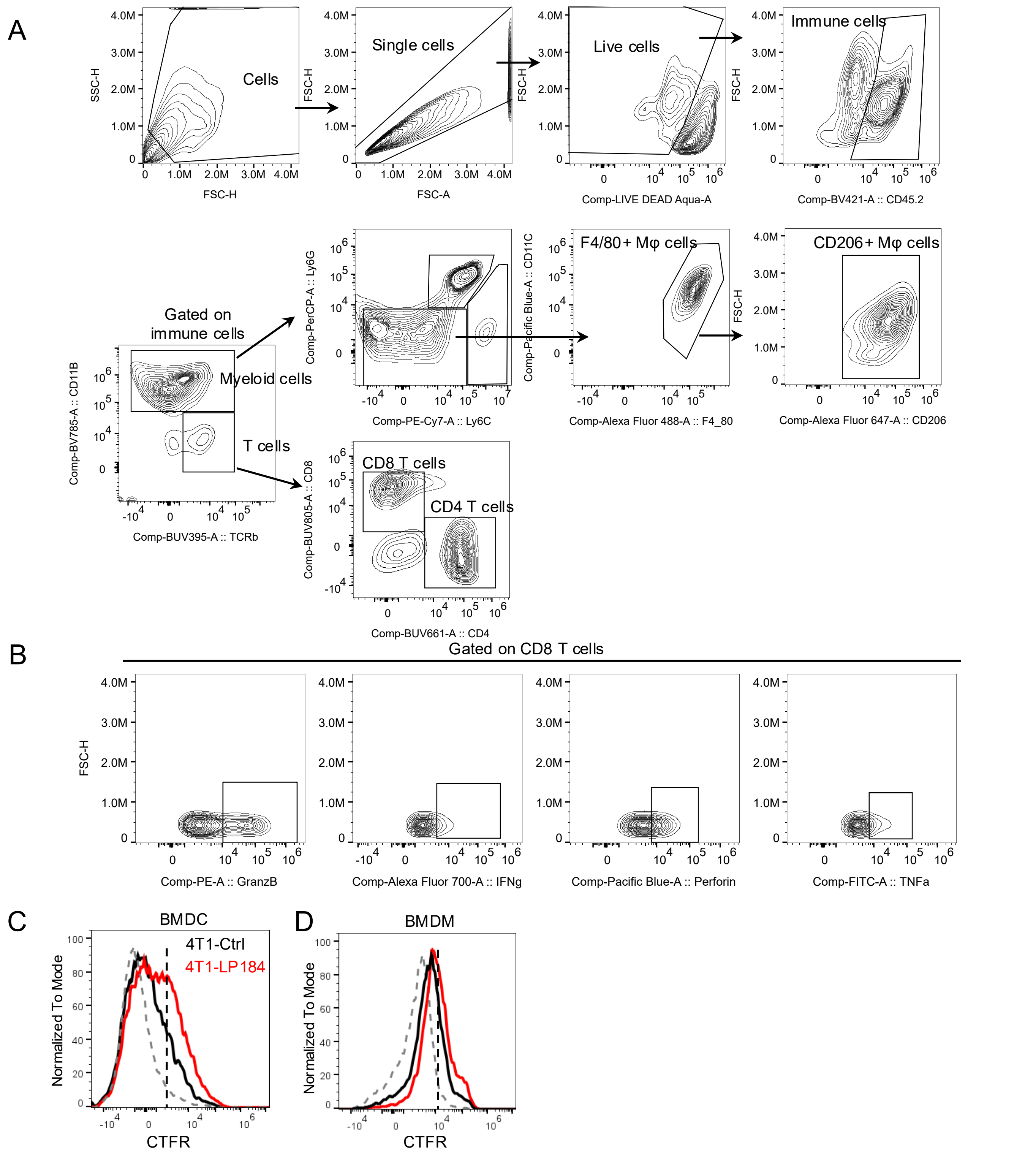

Supplementary Fig.3: Representative flow-cytometric gating strategy for immune cells, cytotoxic molecules and phagocytosis.

A–B, Representative flow-cytometric gating strategy used to quantify immune subsets in T11 tumors. **A,** Sequential gating for live, single, $CD45^+$ immune cells followed by lineage subdivision into myeloid ($CD11b^+$) and T cells ($CD3^+/TCR\beta^+$). $CD206^+$ macrophages were further gated as $Ly6C^-Ly6G^-F4/80^+CD206^+$ within the myeloid population, and T-cell subsets were defined as $CD4^+$ and $CD8^+$ within the T-cell population. **B,** Gating strategy for $CD8^+$ T-cell cytotoxic function showing intracellular staining for Granzyme B, IFN-γ, Perforin, and TNF-α. **C–D,** Representative flow cytometry gating and histograms showing CFTR fluorescence intensity as a measure of phagocytic uptake. (**C**) Bone marrow–derived dendritic cells (BMDCs) and (**D**) bone marrow–derived macrophages (BMDMs) were co-cultured with either CFTR-labeled untreated 4T1 cells or 4T1 cells pretreated with LP-184. Gray: untreated BMDCs/BMDMs alone (control); black: BMDCs/BMDMs co-cultured with untreated 4T1 cells; red: BMDCs/BMDMs co-cultured with LP-184–pretreated 4T1 cells.

Supplementary Fig.4: scRNA-seq reveals that LP-184 + anti–PD-1 remodels the tumor immune ecosystem by reducing suppressive macrophages and enhancing T-cell activation.

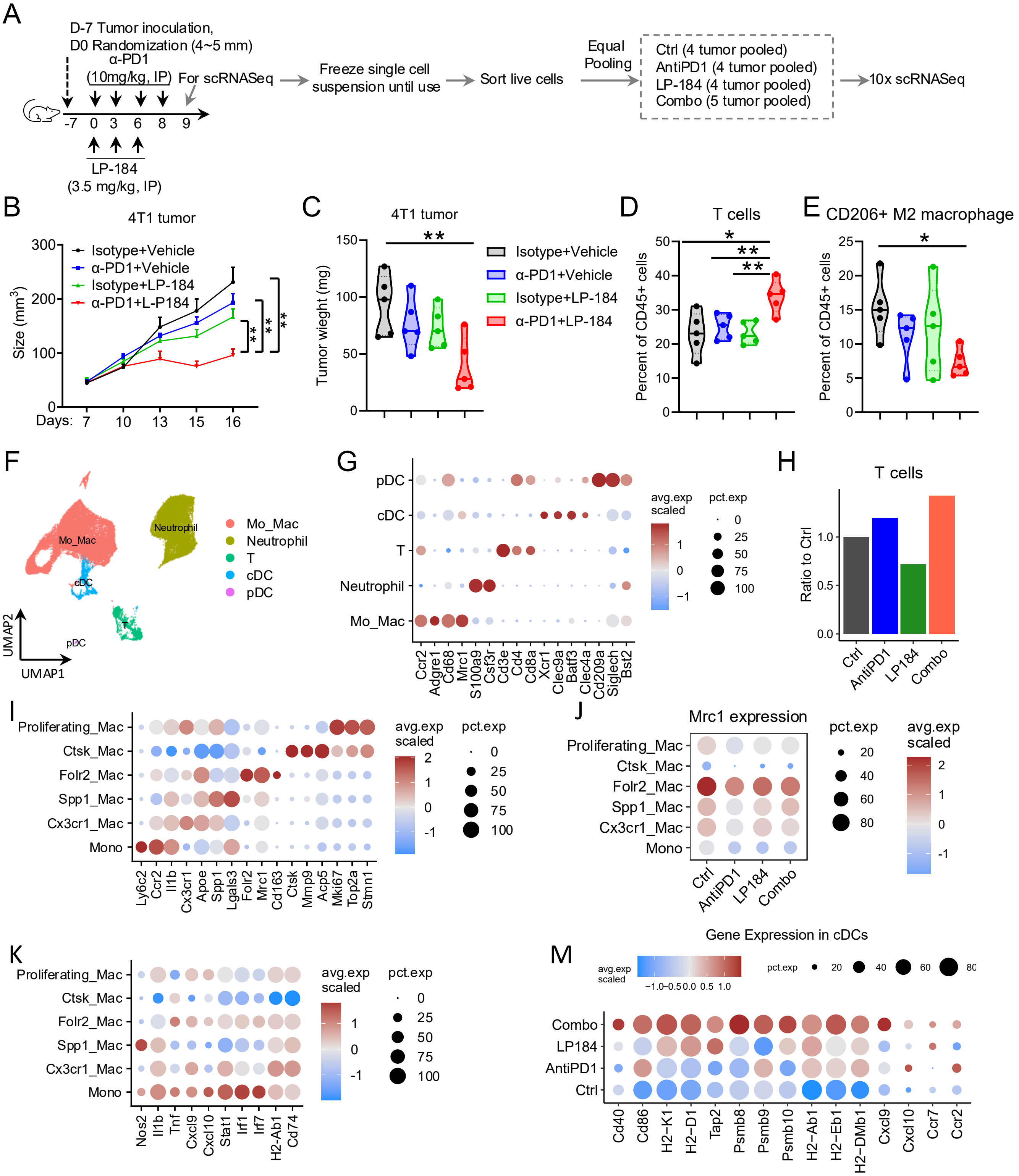

Supplementary Fig.4: scRNA-seq reveals that LP-184 + anti–PD-1 remodels the tumor immune ecosystem by reducing suppressive macrophages and enhancing T-cell activation.

A, Experimental scheme of 4T1 tumor inoculation, treatment, and scRNA-seq library construction and analysis. **B,** Tumor growth curves under indicated treatments. **C,** Tumor weight at endpoint. **D-E,** Flow cytometry analysis of tumor-infiltrating T cells **(D)** and $CD206^{+}$ M2-like macrophages **(E)**. **F,** UMAP of major immune cell lineages in tumors. **G,** Heatmap of gene expression in immune cell clusters. **H,** Relative abundance of T cells normalized to control. **I,** Marker gene expression across macrophage subsets used to define cluster identities. **J,** Mrc1 expression across macrophage subsets and treatment groups. **K,** Expression of inflammatory, interferon-response, and antigen-presentation genes across macrophage subsets. **M,** Gene expression signatures in cDCs, including antigen-presentation and co-stimulatory programs, T-cell–chemotactic chemokines, and dendritic-cell homing receptors. Data represent pooled tumors for each treatment group.
